# Supplementary material for: A molecular toolkit of cross-feeding strains for engineering synthetic yeast communities
Source: Nat Microbiol. 2024 Feb 7;9(3):848–63. doi: 10.1038/s41564-023-01596-4 (PMC10914607; doi:10.1038/s41564-023-01596-4)
Supplement: Supplementary file 1 — Supplementary Notes 1–5 and Figs. 1–33. [file 41564_2023_1596_MOESM1_ESM.pdf]

nature microbiology

Resource

https://doi.org/10.1038/s41564-023-01596-4

A molecular toolkit of cross-feeding strains for engineering synthetic yeast communities

In the format provided by the authors and unedited

Contents

Supplementary Note 1 Initial model analysis and parameter sets 3

Supplementary Note 2 Global sensitivity analysis of the two-strain model 4

Supplementary Note 3 Global sensitivity analysis of the three-strain models 7

Supplementary Note 4 Global sensitivity analysis of the two-member co-culture system with additional pathway burden 11

Supplementary Note 5 Targeted screening using adenine-exchanged metabolite cross-feeding co-cultures 13

Supplementary Figures 14

Fig. S1 The impact of allowing initial population size to vary on the global sensitivity analysis results 14

Fig. S2 The expanded global sensitivity analysis results of the two-member co-culture from Figure 1 of the main text 15

Fig. S3 Full global sensitive analysis of a two-member co-culture accounting for metabolite toxicity 16

Fig. S4. Full global sensitive analysis of a two-member co-culture accounting for exchange metabolite reuptake. 17

Fig. S5 Full global sensitivity analysis of a three-member co-culture with unidirectional metabolite exchange. 18

Fig. S6 Global sensitivity analysis of a three-member co-culture with unidirectional metabolite exchange and with a single reverse exchange due to methionine or tryptophan leak 19

Fig. S7 Global sensitivity analysis of a three-member co-culture with multi-directional metabolite exchange 20

Fig. S8 Global sensitivity analysis of a three-member co-culture with multi-directional metabolite exchange and re-uptake. 21

Fig. S9 Global sensitivity analysis of the division of labour system 22

Fig. S10 Global sensitivity analysis of the division of labour system with key pathway parameters also varied. 23

Fig. S11 Assessment of the expression of three fluorescence proteins on yeast cell growth in different medium. 24

Fig. S12 Validation of reported two pairs of cross-feeding co-cultures. 26

Fig. S13 Cell growth curves of auxotrophic strains with and without amino acid and nucleotide supplementation in synthetic minimal medium. 27

Fig. S14 Time courses of total OD values of adenine-exchanged metabolite I-IV co-cultures in synthetic minimal medium 28

Fig. S15 Time courses of red fluorescent intensities of adenine-exchanged metabolite I-IV co-cultures in synthetic minimal medium 29

Fig. S16 Time courses of blue fluorescent intensities of adenine- exchanged metabolite I-IV co-cultures in synthetic minimal medium 30

Fig. S17 Comparing cell growth and secretion of exchanged metabolites in auxotrophic monocultures that contain an extra-strong or native promoter for exchanged metabolite synthesis........ 32

Fig. S18 Cell growth, exchanged metabolite uptake and secretion of auxotrophic monocultures that contain strong and native promoter for exchanged metabolite synthesis........ 34

Fig. S19 Initial and maximal OD_{700nm} values of two-member and three-member co-cultures and their controls of monocultures and two-member co-cultures within 72 h........ 35

Fig. S20 Promoter engineering controlled cell growth and population size in tryptophan-leucine two-member cross-feeding co-cultures........ 36

Fig. S21 Promoter engineering controlled cell growth and population size in valine - lysine two-member cross-feeding co-cultures........ 37

Fig. S22 Promoter engineering controlled cell growth and population size in tryptophan - lysine two-member cross-feeding co-cultures........ 38

Fig. S23 Promoter engineering controlled cell growth and population size in histidine -lysine two-member cross-feeding co-cultures........ 39

Fig. S24 Effects of metabolite supplementation on cell growth of three pairs of two-member co-cultures........ 40

Fig. S25 Effects of initial cell densities on cell growth and population percentages of three-member co-cultures via two-way communication........ 42

Fig. S26 Effects of initial cell densities on cell growth of three-member co-cultures via two-way communication and their negative controls........ 44

Fig. S27 Time courses of cell growth of four pairs of three-member co-cultures via two-way communication under different initial cell densities........ 45

Fig. S28 Time courses of cell growth of co-culture AKW_VI and its individual member under different initial cell densities........ 46

Fig. S29 Time courses of cell growth of co-culture AKH_VIII and its individual member under different initial cell densities........ 47

Fig. S30 Time courses of cell growth of co-culture AKH_VII and its individual member under different initial cell densities........ 48

Fig. S31 Time courses of cell growth of co-culture AHM_IX and its individual member under different initial cell densities........ 49

Fig. S32 Co-culture strain setup and remaining p-coumaric acid concentrations in synthetic co-cultures for resveratrol production........ 50

Fig. S33 Gating strategy for flow cytometry data........ 51

Supplementary Note 1 Initial model analysis and parameter sets

Note Outline.

Here, analyse the initial simulations to establish how each parameter contributes to a simple two strain co-culture system and describe our global sensitivity analysis methods. We describe the core model derivation, Extended Fourier amplitude global sensitivity analysis in the section of **Online methods** of the main text.

Initial exploration of co-culture design

We first develop a two-member co-culture model which tracks the time evolution of the two strains, denoted A and B. Strain A (B) produces amino acid A (B) and consumes amino B (A). To gain an initial understanding of the impact each parameter has on the dynamics of the system, the final population ratios between the two cultures, and the final population, we parameterise the model as described in the methods and simulate the growth for both individual strains and co-cultures.

In the single strain system, as ϕ_i is increased the growth rate of the strain decreases reducing final population time and increasing batch culture times (**Fig.1, left**). This leads to different timings and magnitude in the peak of amino acid production (**Fig. 1, middle**). The model reveals a nonlinear relationship between growth rate and amino acid production with a peak of amino acid production corresponding to an amino acid production leak of 50% (ϕ_i=0.5) (**Fig. 1, right**).

Simulating the two-strain co-culture shows that high populations are only achieved at intermediate amino acid level production. At $\phi_A > 0.4$ and $\phi_B > 0.4$, amino acid production rates are not sufficient to support good growth of both strains (**Fig. 2, left**). Where there is an asymmetric production rate (e.g. $\phi_A \gg \phi_B$ or vice versa), good growth is obtained as the highly producing strain is able to support large growth of poor producer which in turn generates enough amino acids to support the smaller population of first strain (**Fig. 2, left**). This results in a 'horseshoe' shape where large populations are obtainable at low φ when the values are similar or where there is a large difference between ϕ_A and ϕ_B. As ϕ_A and ϕ_B diverge the population rapidly becomes dominated by the strain which is the poor producer (**Fig. 2, right**).

Nominal parameters. We choose the following nominal parameters: $\gamma_G = 0.05$, $\gamma_i = 0.5$, $V_{max,G}^{y_i} = 7.2$, $K_{M,G}^{y_i} = 5$, $\eta_{y_i} = 0.001$, $V_{max,j}^{y_i} = 30$, $K_{M,j}^{y_i} = 50$, $\delta_i = 1$. These parameters are in the middle of the uptake ranges for the strains in this study (Fig. S17-18). We choose $\phi_i = 0.1$ based on our analysis in Figure 1. We set the initial concentration of glucose $G(t = 0) = 20$ g per L and exchange metabolite $x_i(t = 0) = 0$ mg per L (if absent) or 75 mg per L if present. The initial total population is $N(t = 0) = 0.03$ OD_{600}. The initial population of each species is $y_i(t = 0) = r_{0,i} \cdot N(t = 0)$ where $r_{0,i}$ is the proportion. We simulate all models for a time span of 168 hours.

Supplementary Note 2 Global sensitivity analysis of the two-strain model

Note Outline.

In this note we expand on our analysis in part one of the results by reporting the global sensitivity analysis of the two-member co-culture model.

Part 1. Global sensitivity analysis results with varying initial population size.

We derived the model of the two-strain co-culture system as described in **Supplementary Note** 1. The complete model is shown in Equations 9-13.

$$\frac{dG}{dt} = -J_{upt,G}^{y_1} \cdot y_1 - J_{upt,G}^{y_2} \cdot y_2 \quad (9)$$

$$\frac{dy_1}{dt} = \left(J_{grow}^{y_1} - \eta_{y_1}\right) \cdot y_1 \quad (10)$$

$$\frac{dy_2}{dt} = \left(J_{grow}^{y_2} - \eta_{y_2}\right) \cdot y_2 \quad (11)$$

$$\frac{dx_1}{dt} = J_{leak,1}^{y_1} \cdot y_1 - J_{upt,1}^{y_2} \cdot y_2 \quad (12)$$

$$\frac{dx_2}{dt} = J_{leak,2}^{y_2} \cdot y_2 - J_{upt,2}^{y_1} \cdot y_1 \quad (13)$$

We initially carried out a global sensitivity analysis as outlined in **Supplementary Note 1** allowing all key uptake rates to vary as well as initial population size, initial co-culture composition and metabolite supplementations.

We find that initial population size is the key driver of final population size with eFAST sensitivity of over 0.8, that is 80% of the variation in the final population is due to variation in the starting population N_0 with the remaining 20% shared over the metabolite exchange rates ϕ_1 and ϕ_2 (**Fig. S1A**). Initial population also contributes significantly to the batch culture time (along with glucose uptake rates by the two strains) (**Fig. S1B**). Final population composition is driven by initial composition ($r_{0,1}$ and $r_{0,2}$) as well as glucose uptake parameters (**Fig. S1C, D**). The strain growth rate is driven largely by glucose uptake rate ($V_{max,G}^{y_i}$) with significant contributions from metabolic production (ϕ_i), initial composition and initial population size (**Fig. S1E, F**). We find that in this two-member system, the sensitivities are the same for both strains, e.g., the second most sensitive parameter of y_1 growth rate is ϕ_1 and the second most sensitive parameter of y_2 growth rate is ϕ_2.

Part 2. Global sensitivity analysis results with fixed initial population size (as described in the main text).

Given the significant contribution of initial population size above, we decided to set this parameter constant while enabling the other parameters to vary in the analysis. These results are reported in the main text and are elaborated here. Whilst all parameters increase in their first order sensitivity, we found the order of parameters does not change significantly (comparing, for example, **Fig. S1A** and **Fig. S2A**). This new analysis shows that final population size is most sensitive to the metabolite exchange parameters (ϕ) but relatively insensitive to experimentally tractable parameters such as metabolite supplementation ($x_{0,i}$)) and initial population ratios ($r_{0,i}$) and glucose/metabolite uptake

parameters (i.e., V_{max} parameters) (**Fig. S2A**). Batch culture times are most sensitive to glucose accumulation parameters (with sensitivities of more than 0.2), the next most sensitive parameters are metabolite exchange (**Fig. S2B**). Whilst, total population and batch culture times are insensitive to the initial population ratios, these parameters are key to establishing the final population composition as are the metabolite exchange rates (ϕ_i) (**Fig. S2C, D**). The growth rate of each strain, y_i, is determined primarily by its own glucose assimilation rate (with 50% of the eFAST sensitivity corresponding to $V_{max,G}^{y_i}$). The remaining control of y_i growth rate is shared across the starting population ratios ($r_{0,1}$ and $r_{0,2}$), glucose assimilation of the partner strain (i.e., $V_{max,G}^{y_j}$ where $j \neq i$) and the metabolite production rate ϕ_i, showing again that over production of the exchange metabolite is a key driver of population dynamics. Total metabolite production is determined by glucose assimilation of both strains and by metabolite exchange rate ϕ_i. Total metabolite uptake is largely determine by the initial metabolite concentration and the strain glucose uptake rate suggesting that these may be relatively easy to design due to lack of additional interactions (**Fig. S2G, H, I, J**).

Part 3. Global sensitivity analysis of a two-strain co-culture with toxicity of the metabolites included.

High nucleotide or amino acid concentrations can be toxic to *S. cerevisiae* growth (such as adenine, histidine and lysine, **Fig. S18A, E, H**), in addition to the metabolic burden of metabolite over production. To account for the impact of toxicity of the exchange metabolites on the strain y_i we modify Eq. 6 from **Supplementary Note 1** multiplying the growth rate by a toxicity factor T^{y_i}. We replace Eq.6 with Eq. 14:

$$J_{grow}^{y_i} = \min\left(J_{grow,G}^{y_i}, J_{grow,j}^{y_i}\right) \cdot T^{y_i} \quad (14)$$

The toxicity scaling factor T^{y_i} is

$$T^{y_i} = \frac{1}{\left(1 + \frac{x_i}{\kappa_i^{y_i}} + \sum_{j \neq i}\left(\frac{x_j}{\kappa_j^{y_i}}\right)\right)} \quad (15)$$

where x_i is the metabolite produced by y_i and x_j are the metabolite produced by the other strains in the co-culture. The parameters $\kappa_i^{y_i}$ and $\kappa_j^{y_i}$ are the Michaelis constants governing the impact of the toxicity of x_i on y_i.

In the presence of metabolite toxicity, the most important parameters in determining the final population size are still the metabolite exchange parameters (ϕ_1 and ϕ_2). The parameters governing the impact of toxicity ($I_j^{y_i}$) are next most important in determining maximal population size (**Fig. S3A**). Batch culture times remain largely determined by the glucose accumulation parameters, and the next most sensitive parameters are metabolite exchange (**Fig. S3B**). The final population ratios remain most sensitive to glucose intake rates, and metabolite exchange, with only minor contributions of toxicity parameters (**Fig. S3C, D**). The growth rate of each strain, y_i, is determined primarily by its own glucose assimilation rate and production of its metabolite (**Fig. S3E, F**). Metabolite production and uptake are largely insensitive to the toxicity parameters and follow the same pattern as in Part 2 (**Fig. S3G, H, I, J**).

Part 4 Global sensitivity analysis of a two-strain co-culture with metabolite re-uptake included.

In Parts 1-3, the model was constructed on the assumption that over-producing strains will not take up the exchange metabolite they produce or that any uptake is small and such as a negligible impact on dynamics. For examples, we assumed that exchange metabolite x_1, produced by strain y_1, is only

taken up by y_2 not y_1. However, our preliminary experiments indicated that our over-producing strains consumed the exchange metabolites they produce among different time points, such as strain his+lysΔ took up histidine (1st time point vs 2nd time point), strain leuΔlys+ took up lysine (2nd time point vs 3rd time point) (**Fig. S17 H-J**). To account for metabolite production and (re)uptake we modified the exchange metabolite dynamics (Eq. 12-13) to account for the additional uptake of metabolite x_i by strain y_i.

$$\frac{dx_1}{dt} = J^{y_1}_{leak,1} \cdot y_1 - J^{y_1}_{upt,1} \cdot y_1 - J^{y_2}_{upt,1} \cdot y_2 \tag{16}$$

$$\frac{dx_2}{dt} = J^{y_2}_{leak,2} \cdot y_2 - J^{y_2}_{upt,2} \cdot y_2 - J^{y_1}_{upt,2} \cdot y_1 \tag{17}$$

If the strain is taking up a given metabolite from the culture medium, we assume that it may be growth limiting and so we modify Eq. 6 to account for growth on the produced metabolite as follows:

$$J^{y_i}_{grow} = \min\left(J^{y_i}_{grow,G},\ J^{y_i}_{grow,1},\ J^{y_i}_{grow,2}\right) \tag{18}$$

We repeated our global sensitivity analysis to assess the impact of these additional interactions have on the importance of different engineering strategies. We allowed the parameters of the re-uptake mechanism (i.e. those governing x_i and y_i) to vary in the same way as those governing the uptake of the other exchange metabolites. Our analysis shows that the additional uptake reactions do not significantly change the performance's sensitivities to key engineering strategies. The total population size remains most sensitive to the metabolite exchange parameters (ϕ_1 and ϕ_2) (**Fig. S4A**). Batch culture times remain largely determined by the glucose accumulation parameters, and the next most sensitive parameters are metabolite exchange (**Fig. S4B**). The final population ratio is most sensitive to the glucose intake rates of the two strains and the initial population ratio (**Fig. S4C, D**). The growth rate of each strain, y_i, is determined primarily by the overproduction of its exchange metabolite and own glucose assimilation rate, followed by the glucose consumption rate of the second gene **(Fig. S4E, F)**. Metabolite production and uptake largely follow the same pattern as in Part 2 **(Fig. S4G, H, I, J)**.

Supplementary Note 3 Global sensitivity analysis of the three-strain models

Note Outline.

In this supplementary note we extend our ensemble approach to consider three-member co-cultures with a variety of metabolite exchange mechanisms. In part 1, we consider uni-directional metabolite exchange; for example, strain A (genotype A+/B-) consumes the amino acid produced by strain B (genotype B+/C-) which consumes the amino acid produced by strain C (genotype C+/A-) which consumes the amino acid produced by strain A. Our preliminary experimental results indicated that the native tryptophan and methionine pathways may result in sufficient production of those amino acids, which can be secreted into the culture medium. Therefore, in part 2, we consider uni-directional metabolite exchange but with a third interaction. For example, strain A (genotype A+/B-/C+) secretes metabolites A and C which are consumed by strains B (B+/C-) and C (C+/A-) while strain C also supports the growth of strain B. Finally in Part 3, we consider multi-directional communication, i.e., each strain takes up the amino acids produced by the two other strains and itself produces two amino acids needed by the other strains.

In **Supplementary Note 2**, we showed that the two-member co-culture system's dynamics (population size and batch culture time) were most sensitive to the initial population. When initial population was kept constant in the sensitivity analysis, the magnitude of the sensitivities of the other parameters changed but their order of importance did not. We observe the same result here (not shown for brevity) but choose to focus our discussion on the other system parameters. We keep initial population size constant to allow better resolution of the other sensitivities.

Part 1. Design of a three-member co-culture system with uni-directional communication.

We derived the model of the three-strain co-culture system as described in **Supplementary Note 1** based on the topology shown in **Fig. 5A**. The complete model of the three-member system is depicted in Eq. 19-25.

$$\frac{dG}{dt} = -J_{upt,G}^{y_1} \cdot y_1 - J_{upt,G}^{y_2} \cdot y_2 - J_{upt,G}^{y_3} \cdot y_3 \qquad (19)$$

$$\frac{dy_1}{dt} = \left(J_{grow}^{y_1} - \eta_{y_1}\right) \cdot y_1 \qquad (20)$$

$$\frac{dy_2}{dt} = \left(J_{grow}^{y_2} - \eta_{y_2}\right) \cdot y_2 \qquad (21)$$

$$\frac{dy_3}{dt} = \left(J_{grow}^{y_3} - \eta_{y_3}\right) \cdot y_3 \qquad (22)$$

$$\frac{dx_1}{dt} = J_{leak,1}^{y_1} \cdot y_1 - J_{upt,1}^{y_3} \cdot y_3 \qquad (23)$$

$$\frac{dx_2}{dt} = J_{leak,2}^{y_2} \cdot y_2 - J_{upt,2}^{y_1} \cdot y_1 \qquad (24)$$

$$\frac{dx_3}{dt} = J_{leak,3}^{y_3} \cdot y_3 - J_{upt,3}^{y_2} \cdot y_2 \qquad (25)$$

We carried out a global sensitivity analysis using the methods outlined in **Supplementary Note 1**. For the key co-culture performance metrics (population, batch culture time and final composition) we find large differences between the first-order and total-order effects. This indicates the importance of non-first order effects in the system indicating the significant impact of interactions between the parameters on system behaviour and suggesting these systems are difficult to engineer. The final population size is driven primary by the three metabolite exchange parameters ϕ_1, ϕ_2 and ϕ_3 (**Fig. S5A**). Higher order interactions result in a strong influence of population size of the culture member's glucose update rates ($V_{max,G}$ parameters). The sensitivity analysis suggests that population is not significantly affected by metabolite uptake rate ($V_{max,i}$), initial starting population ($r_{0,i}$) or metabolites supplementations ($x_{0,i}$). Batch culture time is primarily driven by glucose uptake rate parameters which are difficult to engineer *in vivo*. The engineerable parameters (metabolite exchange rate, starting composition and metabolite supplementation) share most of the responsibility equally for the variance in batch culture time (**Fig. S5B**). The final culture composition (y_i ratio) is driven primarily by the glucose uptake parameters of all three strains (i.e., strain y_1 is affected by those of strains 1, 2 and 3) (**Fig. S5C, D, E**). Its own starting proportion and strength of its own metabolite production are key secondary influences. The impact of metabolite exchange (ϕ parameter) is the second most important determinant of strain growth rate (**Fig. S5F, G, H**).

Part 2. Design of a three-member co-culture system with uni-directional communication and a second metabolite exchange reaction.

We modified the model of the three-member co-culture with uni-directional metabolite exchange (Eq. 19-25) to account for the unanticipated second metabolite exchange which occurs in some strains due to natural metabolite secretion. We update the model topology by assuming that strain y_1 also produces metabolite x_3 at a rate determined by $\phi_{3,1}$. We modified Eq. 25 to account for the additional production dynamics of x_3:

$$\frac{dx_3}{dt} = J_{leak,3}^{y_3} \cdot y_3 + J_{leak,3}^{y1} - J_{upt,3}^{y_2} \cdot y_2 \tag{26}$$

To account for the additional glucose diversion to x_3 rather than biomass of strain y_1 we modified the growth rate (Eq 6.) as follows:

$$J_{grow,G}^{y_i} = \gamma_G \cdot (1 - \phi_i - \phi_j) \cdot J_{upt,G}^{y_i} \tag{12}$$

We repeated our global sensitivity analysis for this new model (**Fig. S6**). Whilst the results broadly replicate those from part 1 of this supplementary note there are key minor differences. The system's final population becomes more sensitive to ϕ_2 and ϕ_3 (i.e., metabolite production from the non-leaky strains) and to the glucose uptake rates (**Fig. S6A**). The ϕ_1 falls from joint first place in the sensitivity ranking to sixth. The sensitivity of the batch culture time remains determined by the glucose uptake of strains (**Fig. S6B**). The sensitivity of the final population composition (the y_1, y_2 and y_3 ratios) changes with the final population of y_1 becoming more sensitive to metabolite exchange than the starting conditions of the other two strains (**Fig. S6C**). The strains which have only one metabolite exchange (y_2 and y_3) show similar results to those in part 1 but with first-order sensitivities falling; showing that variance in this performance metric is spread out over other parameters (**Fig. S6D, E**).

Part 3. Design of a three-member co-culture system with multi-directional communication.

We next considered the case of a three-member co-culture with multi-directional communication, i.e., each strain takes up the exchange metabolites produced by the two other strains rather than

just one (see **Fig. 5D** for an experimental example). Therefore, we updated Eq. 23-25 to account for the additional metabolite uptake of the second strain:

$$\frac{dx_1}{dt} = J_{leak,1}^{y_1} \cdot y_1 - J_{upt,1}^{y_2} \cdot y_2 - J_{upt,1}^{y_3} \cdot y_3 \quad (28)$$

$$\frac{dx_2}{dt} = J_{leak,2}^{y_2} \cdot y_2 - J_{upt,2}^{y_1} \cdot y_1 - J_{upt,2}^{y_3} \cdot y_3 \quad (29)$$

$$\frac{dx_3}{dt} = J_{leak,3}^{y_3} \cdot y_3 - J_{upt,3}^{y_1} \cdot y_1 - J_{upt,3}^{y_2} \cdot y_2 \quad (30)$$

We modified the growth definition $J_{grow}^{y\_i}$ to account for growth on the second metabolite x_j as follows:

$$J_{grow}^{y_i} = \min\left(J_{grow,G}^{y_i}, \ J_{grow,i}^{y_i}, \ J_{grow,j}^{y_i} \right) \quad (31)$$

We find that initial population size is the key driver of the final population size with eFAST sensitivity of over 0.9, that is 90% of the variation in the final population is due to variation in the starting population. Therefore, we fixed the starting population and allowed the other parameters (shown in **Fig. S7**) to vary. Final population size sensitive to metabolite exchange between two of the strains only, i.e., ϕ_1 and ϕ_2 each have sensitivities over 0.1 with ϕ_3 having only a negligible impact. Note that these sensitivities are low with multiple parameters contributing population size (**Fig. S7A**). Glucose assimilation rate is a key driver of population size and batch culture time (**Fig. S7A, B**). The final composition is driven by glucose uptake rates of two of the strains (1 and 2 while the third is free to vary) (**Fig. S7C, D, E**). Fine engineering of glucose uptake rate is difficult to achieve experimentally, but our analysis shows that varying the initial starting proportion of these strains ($r_{0,i}$) and their metabolite exchange parameters also has significant impact on final composition, with the initial composition having slightly more impact. Our analysis suggests that fine control of the system can be achieved through careful control of two strains while the third design can be less certain/characterised.

Part 4. Design of a three-member co-culture system with multi-directional communication and exchange metabolite re-uptake

To explore the impact of exchange metabolite uptake by the production strain (e.g. the uptake of x_1 by y_1), we updated the metabolite dynamics by replacing Eq. 28-30 with Eq. 32-34 as follows:

$$\frac{dx_1}{dt} = J_{leak,1}^{y_1} \cdot y_1 - J_{upt,1}^{y_1} \cdot y_1 - J_{upt,1}^{y_2} \cdot y_2 - J_{upt,1}^{y_3} \cdot y_3 \quad (32)$$

$$\frac{dx_2}{dt} = J_{leak,2}^{y_2} \cdot y_2 - J_{upt,2}^{y_1} \cdot y_1 - J_{upt,2}^{y_2} \cdot y_2 - J_{upt,2}^{y_3} \cdot y_3 \quad (33)$$

$$\frac{dx_3}{dt} = J_{leak,3}^{y_3} \cdot y_3 - J_{upt,3}^{y_1} \cdot y_1 - J_{upt,3}^{y_2} \cdot y_2 - J_{upt,3}^{y_3} \cdot y_3 \quad (34)$$

If the strain is taking up a given metabolite from the culture medium, we assume that it may be growth limiting and so we modify $J_{grow}^{y\_i}$ to account for growth on the produced metabolite as follows:

$$J_{grow}^{y_i} = \min(J_{grow,G}^{y_i}, \ J_{grow,1}^{y_i}, \ J_{grow,2}^{y_i}, \ J_{grow,3}^{y_i}) \quad (35)$$

We subjected this new model to global sensitivity analysis as before, again fixing the total initial population size. The addition of the metabolite reuptake reactions reduced the sensitivities of each performance metric to each parameter making the identification of key "engineering dials" more complex. We find that final population is largely driven by metabolite production rates (ϕ_i) and initial metabolite concentration ($x_{0,i}$) (**Fig. S8A**). Although the first order sensitivities are low and the high total order sensitivities of all the parameters shows the control of final population is distributed across all parameters. Total batch culture time is also distributed across all parameters with all first order sensitivities below 0.1 (**Fig. S8B**). The composition of the culture (i.e. the y_1, y_2 and y_3 ratios) is largely determined by the glucose assimilation rates ($V_{max,G}$) of the three strains. The next most important parameters are the starting ratios ($r_{0,i}$) and production rate of the exchange metabolite (e.g. ϕ_1 for y_1 etc) (**Fig. S8C,D,E**). The growth rate of each strain is largely determined by its glucose assimilation rate (**Fig. S8F, G, H**). The exchange metabolite production rate is determined by ϕ_i, the starting ratio for production strain (i.e. $r_{0,i}$ for strain y_i) and the maximum glucose assimilation rate of each strain (**Fig. S8I, J, K**). The global sensitivity shows the uptake rate is largely driven by the initial exchange metabolite concentration in the supplemented medium (**Fig. S8L, M, N**).

Supplementary Note 4 Global sensitivity analysis of the two-member co-culture system with additional pathway burden

Note Outline.

In this note, we first develop a model of a division of labour system by expanding the two-member model from **Supplementary Note 1** to include production of pathway metabolites. In part 2 we nominally parameterise this model and carry out a global sensitivity analysis. In part 3, we repeat the global sensitivity analysis considering variation in the pathway parameters.

Part 1. Division of labour model.

We introduced two new species into the model z_1 and z_2 which represent p-coumaric acid and resveratrol respectively. The metabolite z_1 is produced from glucose by strain y_1 at rate $J_{G\to z_1}$. Resveratrol, z_2, is produced by strain y_2 from z_1 at rate $J_{z_1\to z_2}$. The dynamics of the pathway metabolites are:

$$\frac{dz_1}{dt} = y_1 \cdot J_{G\to z_1} - y_2 \cdot J_{z_1\to z_2} \tag{36}$$

$$\frac{dz_2}{dt} = y_2 \cdot J_{z_1\to z_2} \tag{37}$$

We assume that z_1 is produced in proportion to the glucose uptake, where δ_{eng} is the number of glucose molecules consumed per z_1 produced and ϕ_{eng} is the proportion of glucose flux diverted to z_1:

$$J_{G\to z_1} = \delta_{eng} \cdot \phi_{eng} \cdot J_{upt,G}^{y_1} \tag{38}$$

We model the conversation of z_1 into z_2 using Michalis-Menten kinetics:

$$J_{z_1\to z_2} = \frac{v_{max,z_1} \cdot z_1}{k_{M,z_1} + z_1} \tag{39}$$

The glucose-limited growth rate of y_1 is modified to take account of the burden due to glucose flux diversion to the new product:

$$J_{grow,G}^{y_1} = \gamma_G \cdot (1 - \phi_1 - \phi_{eng}) \cdot J_{upt,G}^{y_1} \tag{40}$$

To assess the performance of the pathway model we introduce two new metrics: volumetric productivity (which governs production times and is a key driver of process costs in industry) and yield (which governs over all process efficiency). Pathway productivity is calculated by

$$prod = \frac{z_2(t = t_{batch})}{t_{batch}} \tag{41}$$

Where t_{batch} is the length of the batch culture time and is determined as the time point at which $G(t) = 0$. We define the yield as:

$$yield = \frac{z_2(t = t_{end})}{G(t = 0)} \tag{42}$$

Part 2. Global sensitivity analysis results of the division of labour system (as described in the main text).

We carried out a global sensitivity analysis of the new division of labour model from part one using the same methods described previously. The additional interactions, e.g., the presence of the glucose draining metabolic pathway in the z_1 producing strain, resulted in fundamental rearrangements of the parameter sensitivities. In the presence of the metabolic engineered pathway, the sensitivity of population size to ϕ_1 decreases significantly (comparing **Fig. S2A** with **Fig. S9A**). In this system, key drivers of population size is ϕ_2 (with sensitivity of more than 30%) were glucose uptake parameters and initial population composition (**Fig. S9A**). Batch culture times became more sensitive to the glucose assimilation rate of the second strain y_2 (i.e., that which converts z_1 to z_2) (**Fig. S9D**). The final population composition is most sensitive to the glucose assimilation rate of strain y_2 and ϕ_1 (**Fig. S9B, C**) with initial population composition also making a larger contribution than in the original two-member analysis. System productivity and yield show similar sensitivities (**Fig. S9G, J**) with both measures of performance being driven by glucose uptake by both stains, initial population composition, and y_1 to y_2 metabolite exchange strength. Glucose to z_1 is driven largely by the strain's glucose uptake rate (**Fig. S9M**) while the z_1 to z_2 conversion is driven by the key population determining parameters (i.e., the glucose uptake rates of both strains, their initial starting ratio, and ϕ_1) (**Fig. S9N**). This analysis suggests that co-culture dynamics and productivity of the division of labour system can be tuned by engineering the initial population composition.

Part 3. Global sensitivity analysis with key pathway parameters also varied.

We repeated our analysis enabling key pathway parameters, such as ϕ_{eng} and V_{max,z_1}, to also vary. This establishes if interactions between the co-culture control system and pathway dynamics results in changes to the suggested key design parameters. This analysis shows that the burden associated with z_1 production by strain y_1 is a significant determinant of both population size, productivity and yield (**Fig. S10A, G, J**). This added interaction does not significantly re-order the parameter sensitivities, but it does result in increasing sensitivity to ϕ_1 and reduced sensitivity to the starting populations $r_{0,1}$ and $r_{0,2}$ (e.g., **Fig. S10B, C**). This indicates that whilst initial population composition is likely key to establishing the dynamics of the division of labour system, the creation of such systems will require bespoke tuning based on specific pathway burden.

Supplementary Note 5 Targeted screening using adenine-exchanged metabolite cross-feeding co-cultures

In this note, we screened essential metabolite targets for co-culture potential using 52 pairs of adenine-exchanged metabolite (em) cross-feeding co-cultures. In order to test the capacity to establish co-cultures of the new targets and newly generated strains, we decided to pair them independently with an adenine receiver strain. We validated two pairs of co-cultures (ade-lys and trp-leu) in the preliminary experiments (**Fig. S12**), we found that the ade-lys co-culture showed higher growth than the trp-leu co-culture. We then used the ade and lys strains as a reference point—establishing new co-cultures where at least one constituent member of the community had a precedence of forming strong cross-feeding interactions. We then paired the RFP tagged ade auxotrophic strain with strains for other potential targets **(Tab. S1).** For each target metabolite, four pairs of co-cultures were created, which were named ade-em I, II, III, IV (where em stands for exchanged metabolite): ade-em I, overexpression of em and ade in each member; ade-em II, overexpression of ade only in the BFP tagged member; ade-em III, overexpression of em only in the RFP tagged member; ade-em IV, no overexpression in either member **(Extended Data Fig. 1A).** The strong and constitutive promoter pCCW12 was used for overexpression in both strains.

52 pairs of ade-em two-member cross-feeding co-cultures were created for metabolites including arginine (arg), cysteine (cys), histidine (his), leucine (leu), lysine (lys), methionine (met), phenylalanine (phe), tryptophan (trp), tyrosine (tyr), serine (ser), threonine (thr), uracil (ura), valine (val), and isoleucine (ile) **(Fig. 2A)**. The success of these targets for use in co-cultures was determined by their capacity to help the co-cultures grow (i.e., OD) more than their corresponding monocultures controls. As expected, all monoculture controls showed lower growth than the positive controls (prototroph strain) (**Extended Data Fig. 1B**). Although the overexpression of certain targets resulted in an increase of metabolite levels detected by LC-MS (specifically ade, his, lys, phe, tyr, thr, and trp), this effect was not observed in another subset of targets: cys, leu, met, ser, ura, val, and ile (**Fig. S17**). Beyond the ade-arg I-IV and ade-ser I-IV co-cultures that did not grow, most co-cultures did show higher growth than the monoculture controls, especially co-culture ade-aa I, II (**Extended Data Fig. 1C-D**). Moreover, some ade-em I co-cultures grew more than ade-em II, such as ade-his, ade-lys, ade-phe&tyr, ade-thr, ade-trp, which correlated with LC-MS data showing that overexpression produced higher levels of metabolites (**Fig. S17**) to support the growth of the BFP tagged auxotrophic member. Most auxotrophic co-cultures did not grow without gene overexpression (ade-aa IV), which indicated the importance of expressing metabolic genes at sufficient levels to provide ample metabolites for co-culture growth. The majority of ade-em III, IV co-cultures without *ADE4op* expression showed either weak or no growth compared to monocultures (**Extended Data Fig. 1C-D**). The growth dynamics of each member and total population for these co-cultures ade-em I-IV within 72 h can be found in **Fig. S14-16**. Based on the growth (OD_{700nm}) of ade-em I II co-cultures, we classified each target metabolite by their ability to facilitate growth in cross-feeding co-cultures: strong ($OD_{700nm} \geqslant 0.5$): ade, trp, met, his; medium ($0.3 \leqslant OD_{700nm} < 0.5$): lys, phe&tyr, val&ile, cys, leu, ura; and weak ($OD_{700nm} < 0.3$): thr, tyr, arg, ser. In some cases, in the tested conditions (ade-his, ade-lys, ade-phe&tyr, ade-thr, ade-trp), the over-expression of the target metabolite improved the co-culture growth as hypothesised (**Extended Data Fig. 1**). We then performed LC-MS and found that the overexpression of the target genes selected for the overproduction of ade, his, lys, phe, tyr, trp, and thr enhanced their production, which explains our co-culture results (**Fig. S17**). Thus, the molecular toolkit includes three fluorescence proteins, 15 auxotrophic strains (13 presenting strict auxotrophic phenotypes in the tested conditions) and 15 metabolite target genes (7 enhancing production when overexpressed in the tested conditions).

Supplementary Figures

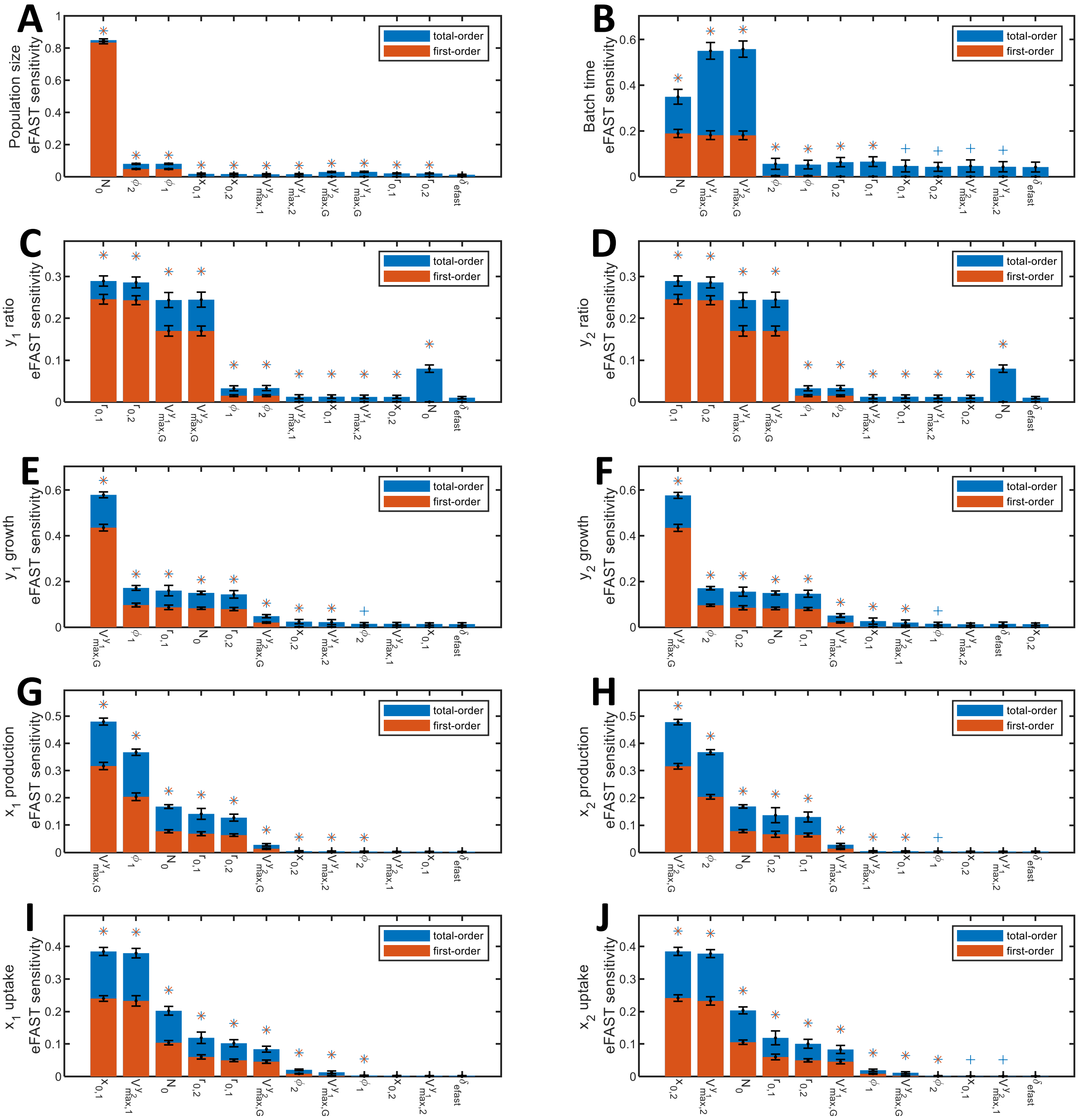

Fig. S1 The impact of allowing initial population size to vary on the global sensitivity analysis results. The global sensitivity analysis was carried out as described in **Supplementary Note 1.** Full results are shown from each performance metric. Orange bars represent the first order sensitivities. Blue bars represent the total order sensitivities. 100 re-samplings were carried out with the mean and standard deviation in the sensitivity reported. Statical significance of the sensitivities relative to the dummy parameter $\delta efast$ was calculated using t-test with an α value of 0.01 adjusted using Bonferroni correction by the number of parameters being tested. "+", sensitivity is significantly different ($p < 0.01$) from the dummy parameter. "x", the total sensitivity is significantly different ($p < 0.01$) from the dummy parameter.

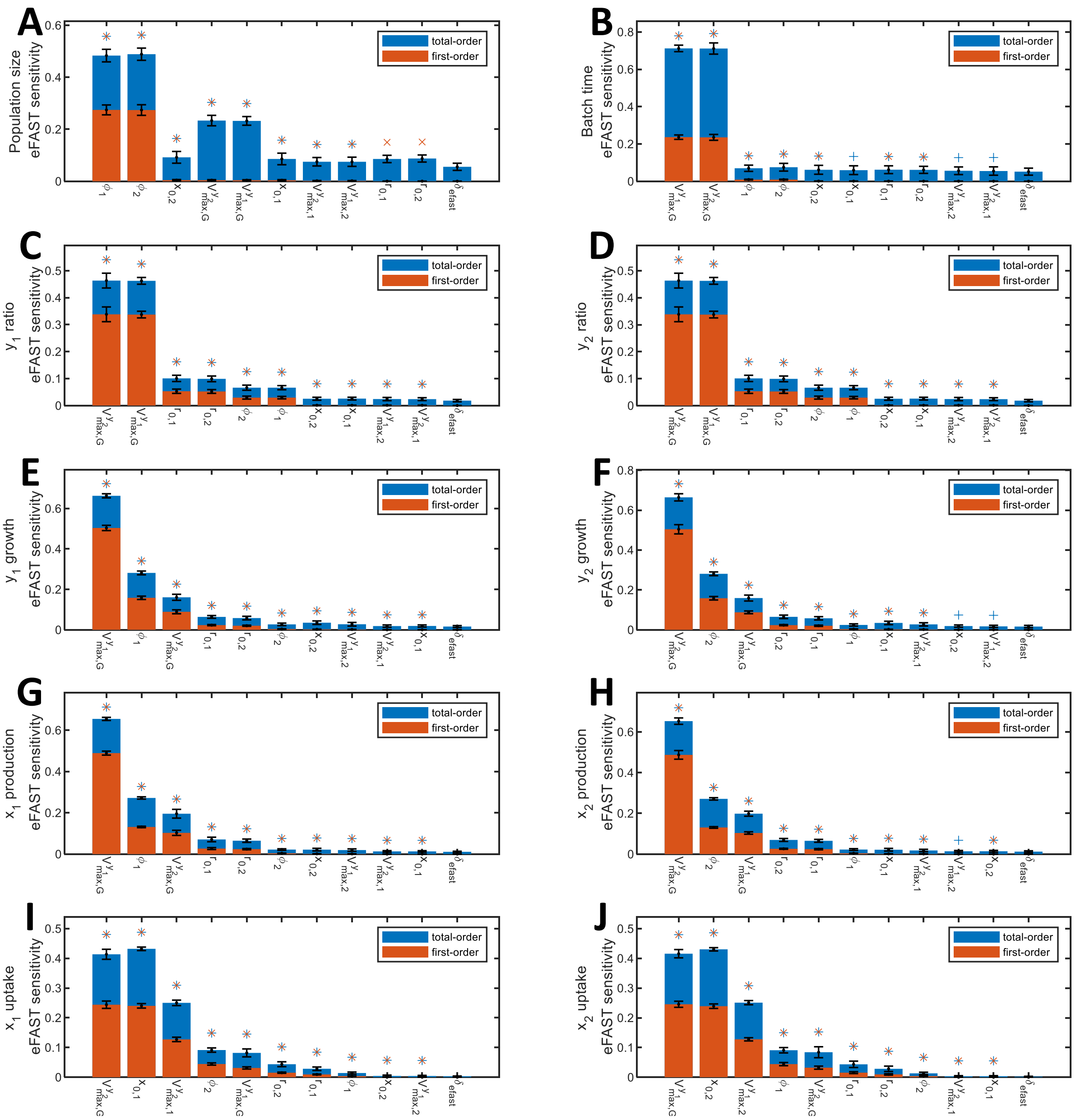

Fig. S2 The expanded global sensitivity analysis results of the two-member co-culture from Figure 1 of the main text.

The global sensitivity analysis was carried out as described in **Supplementary Note 1.** Full results are shown from each performance metric. Orange bars represent the first order sensitivities. Blue bars represent the total order sensitivities. 100 re-samplings were carried out with the mean and standard deviation in the sensitivity reported. Statical significance of the sensitivities relative to the dummy parameter $\delta efast$ was calculated using t-test with an α value of 0.01 adjusted using Bonferroni correction by the number of parameters being tested. "+", sensitivity is significantly different ($p < 0.01$) from the dummy parameter. "x", the total sensitivity is significantly different ($p < 0.01$) from the dummy parameter.

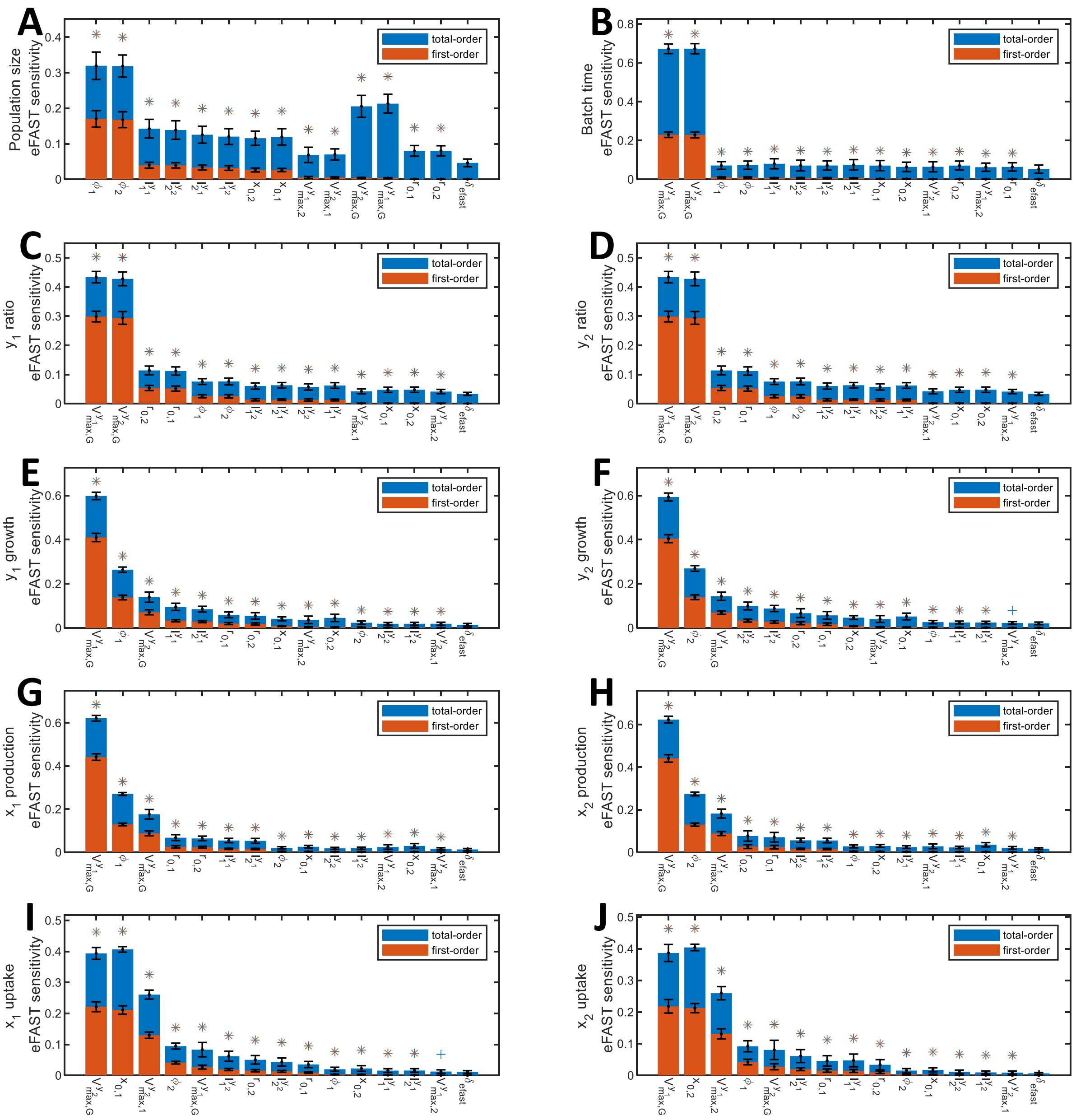

Fig. S3 Full global sensitive analysis of a two-member co-culture accounting for metabolite toxicity. The global sensitivity analysis was carried out as described in **Supplementary Note 1**. Full results are shown from each performance metric. Orange bars represent the first order sensitivities. Blue bars represent the total order sensitivities. 100 re-samplings were carried out with the mean and standard deviation in the sensitivity reported. Statical significance of the sensitivities relative to the dummy parameter $\delta efast$ was calculated using t-test with an α value of 0.01 adjusted using Bonferroni correction by the number of parameters being tested. "+", sensitivity is significantly different ($p < 0.01$) from the dummy parameter. "x", the total sensitivity is significantly different ($p < 0.01$) from the dummy parameter.

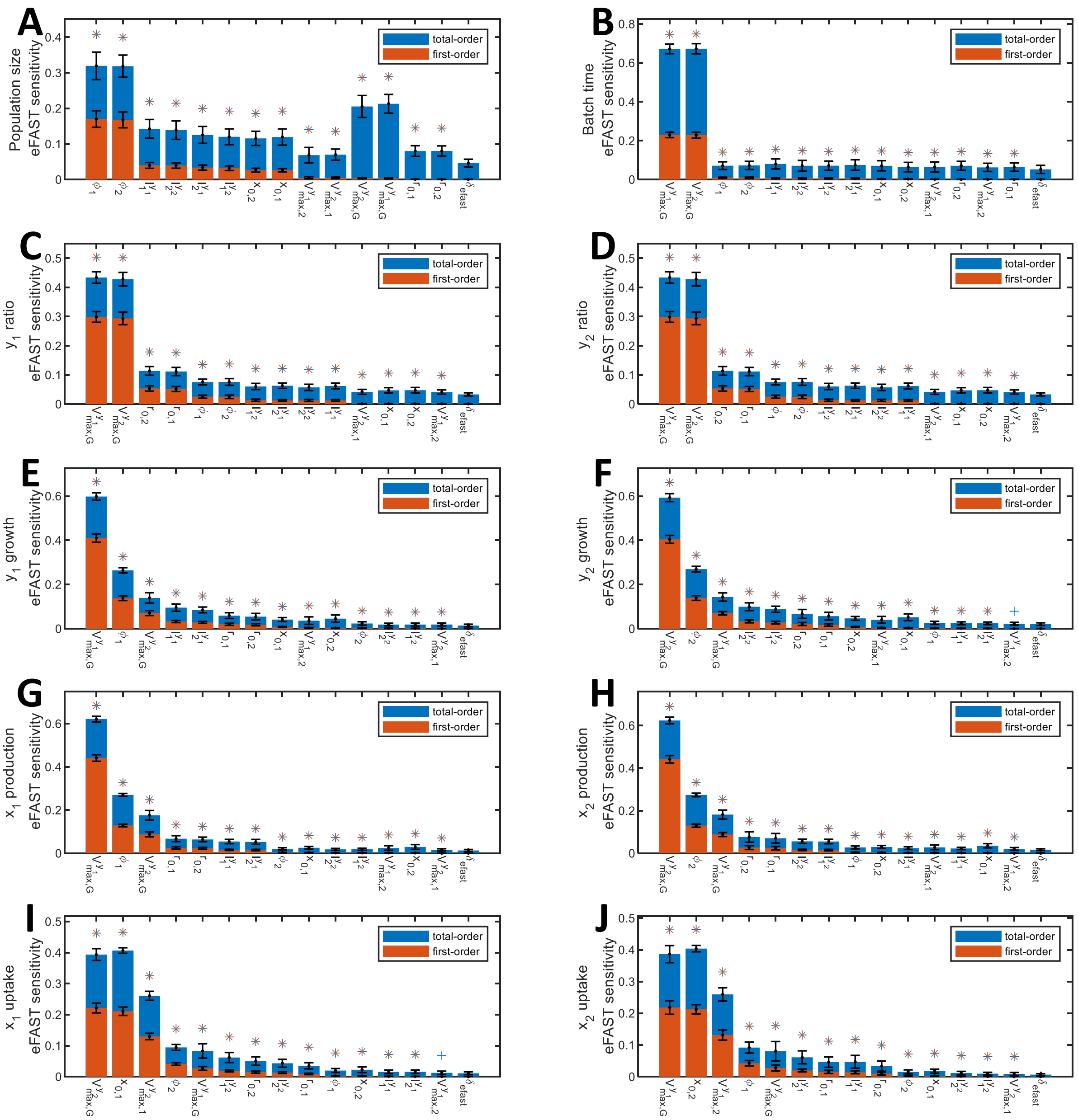

Fig. S4. Full global sensitive analysis of a two-member co-culture accounting for exchange metabolite reuptake.

The global sensitivity analysis was carried out as described in **Supplementary Note 1**. Full results are shown from each performance metric. Orange bars represent the first order sensitivities. Blue bars represent the total order sensitivities. 100 re-samplings were carried out with the mean and standard deviation in the sensitivity reported. Statical significance of the sensitivities relative to the dummy parameter $\delta efast$ was calculated using t-test with an α value of 0.01 adjusted using Bonferroni correction by the number of parameters being tested. "+", sensitivity is significantly different ($p < 0.01$) from the dummy parameter. "x", the total sensitivity is significantly different ($p < 0.01$) from the dummy parameter.

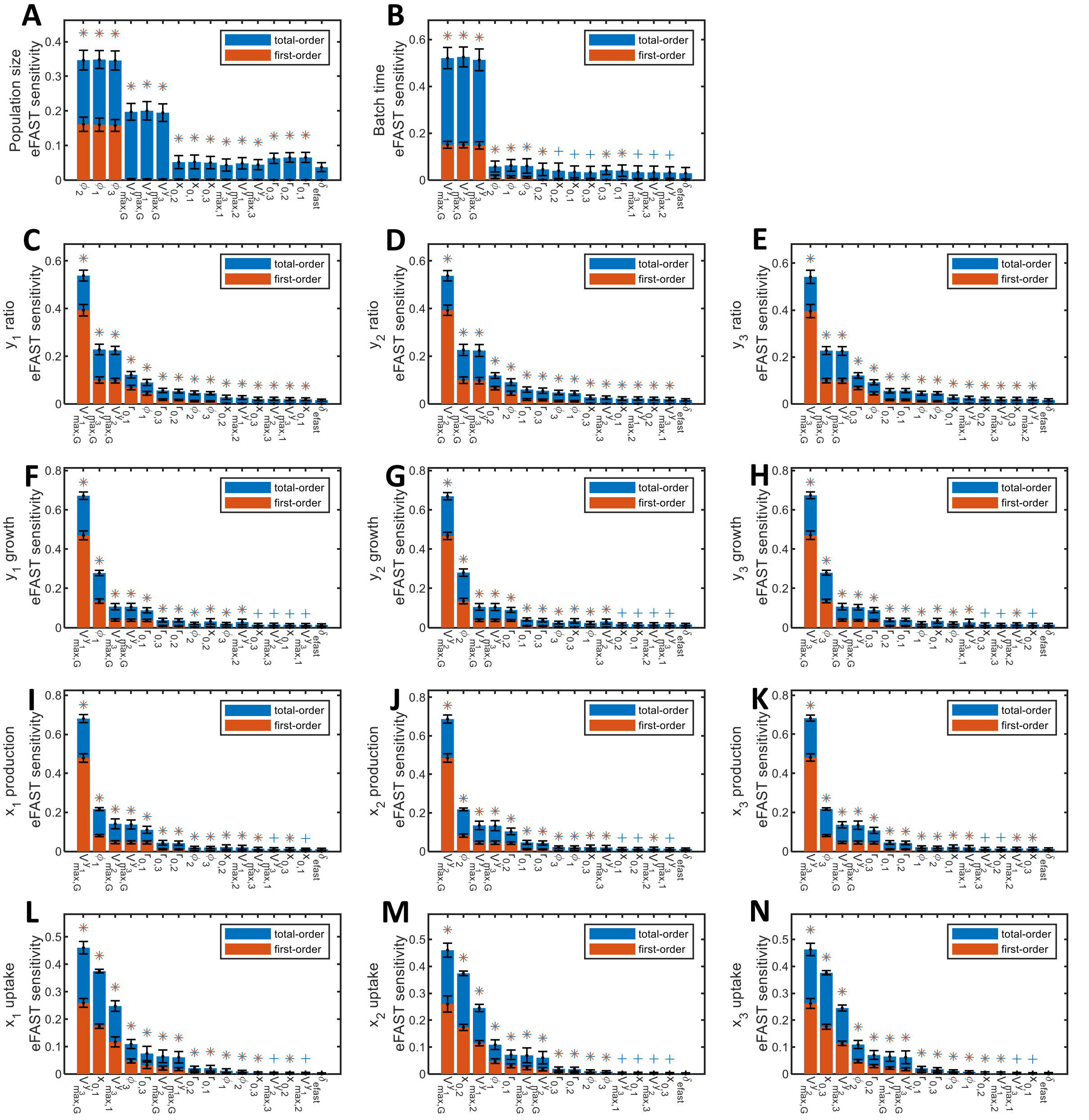

Fig. S5 Full global sensitivity analysis of a three-member co-culture with unidirectional metabolite exchange.

The global sensitivity analysis was carried out as described in **Supplementary Note 1.** Full results are shown from each performance metric. Orange bars represent the first order sensitivities. Blue bars represent the total order sensitivities. 100 re-samplings were carried out with the mean and standard deviation in the sensitivity reported. Statical significance of the sensitivities relative to the dummy parameter $\delta efast$ was calculated using t-test with an α value of 0.01 adjusted using Bonferroni correction by the number of parameters being tested. “+”, sensitivity is significantly different ($p < 0.01$) from the dummy parameter. “x”, the total sensitivity is significantly different ($p < 0.01$) from the dummy parameter.

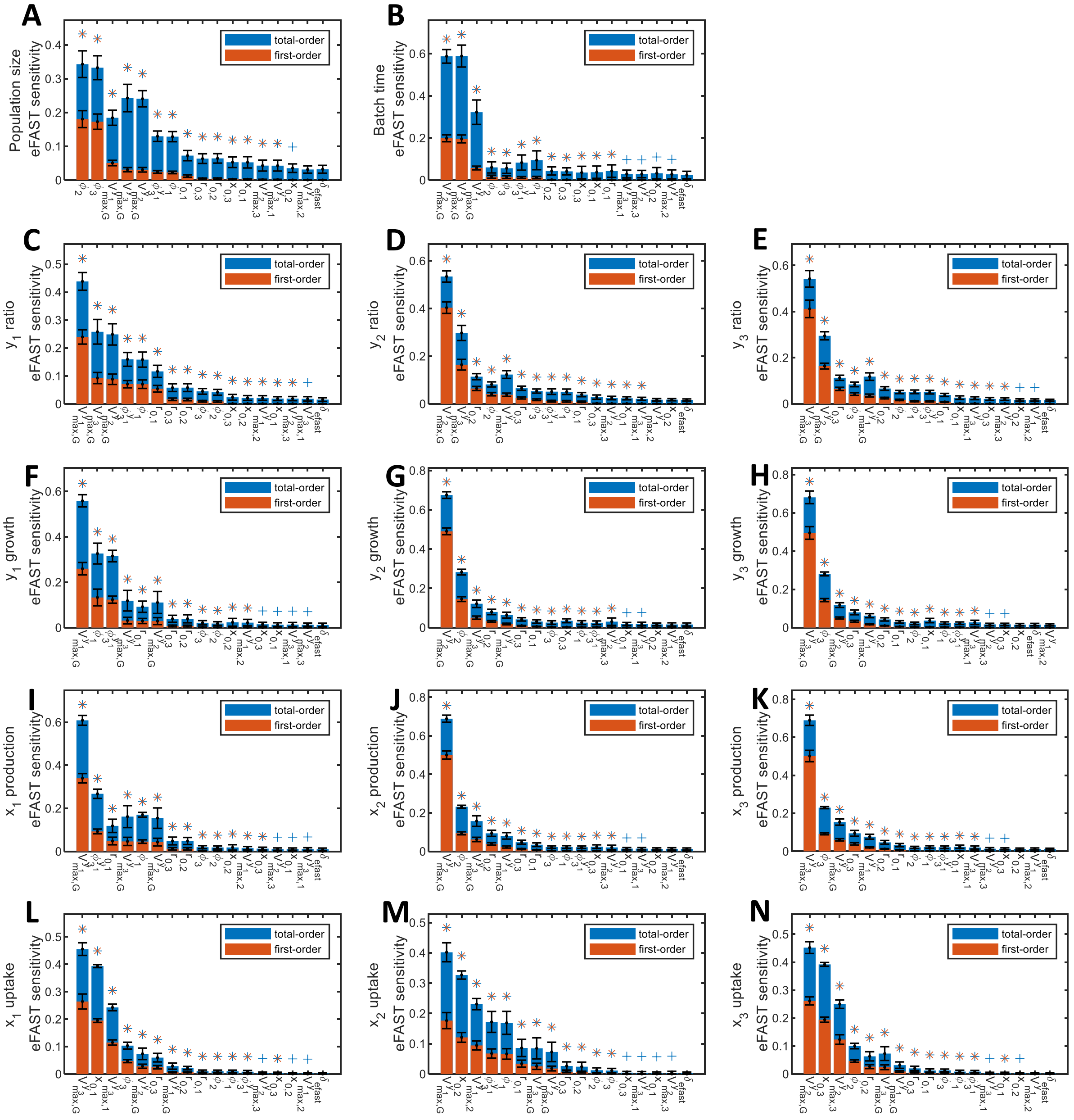

Fig. S6 Global sensitivity analysis of a three-member co-culture with unidirectional metabolite exchange and with a single reverse exchange due to methionine or tryptophan leak.

The global sensitivity analysis was carried out as described in **Supplementary Note 1.** Full results are shown from each performance metric. Orange bars represent the first order sensitivities. Blue bars represent the total order sensitivities. 100 re-samplings were carried out with the mean and standard deviation in the sensitivity reported. Statical significance of the sensitivities relative to the dummy parameter $\delta efast$ was calculated using t-test with an α value of 0.01 adjusted using Bonferroni correction by the number of parameters being tested. “+”, sensitivity is significantly different ($p < 0.01$) from the dummy parameter. “x”, the total sensitivity is significantly different ($p < 0.01$) from the dummy parameter.

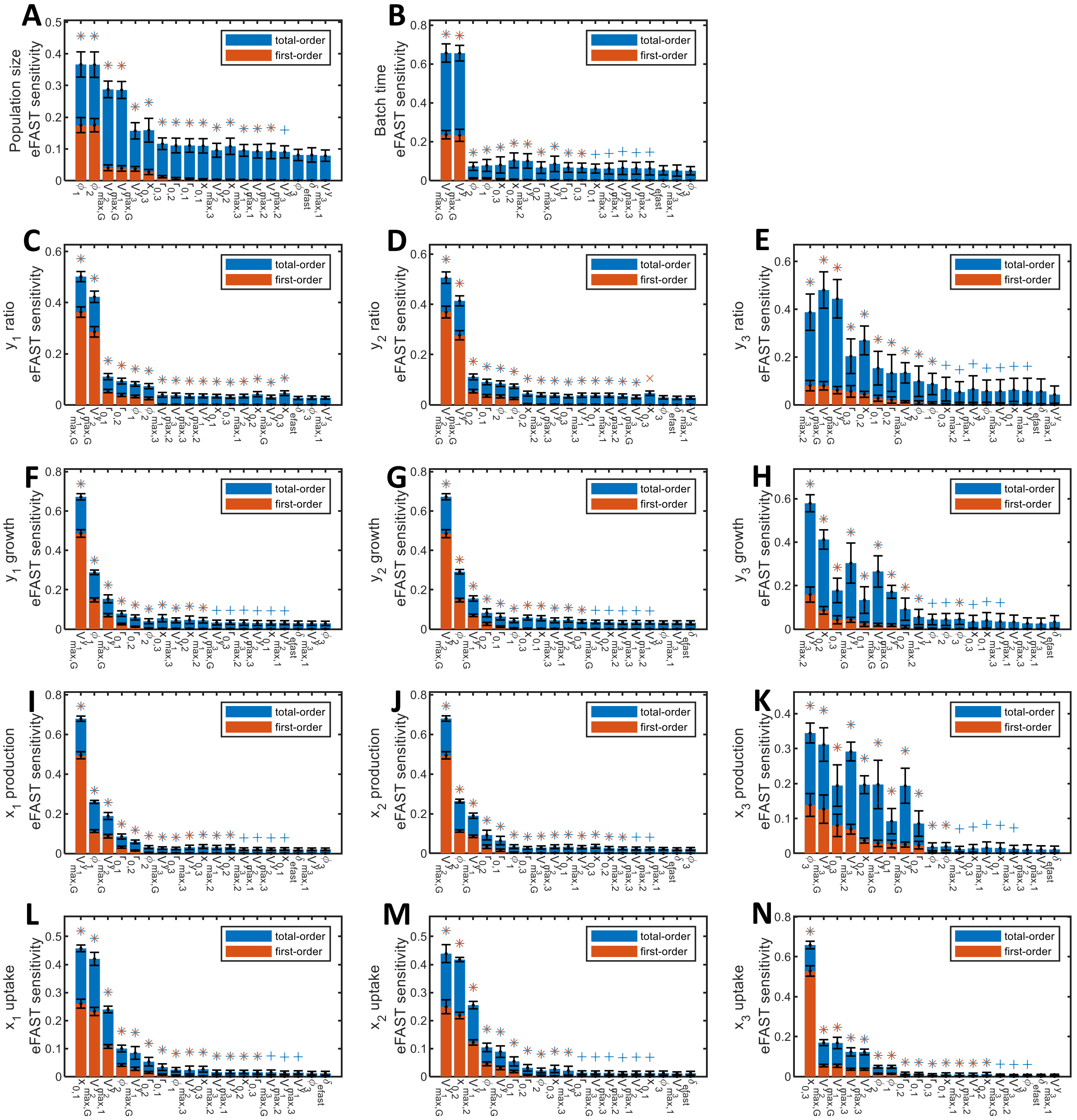

Fig. S7 Global sensitivity analysis of a three-member co-culture with multi-directional metabolite exchange

The global sensitivity analysis was carried out as described in **Supplementary Note 1.** Full results are shown from each performance metric. Orange bars represent the first order sensitivities. Blue bars represent the total order sensitivities. 100 re-samplings were carried out with the mean and standard deviation in the sensitivity reported. Statical significance of the sensitivities relative to the dummy parameter $\delta efast$ was calculated using t-test with an α value of 0.01 adjusted using Bonferroni correction by the number of parameters being tested. “+”, sensitivity is significantly different ($p < 0.01$) from the dummy parameter. “x”, the total sensitivity is significantly different ($p < 0.01$) from the dummy parameter.

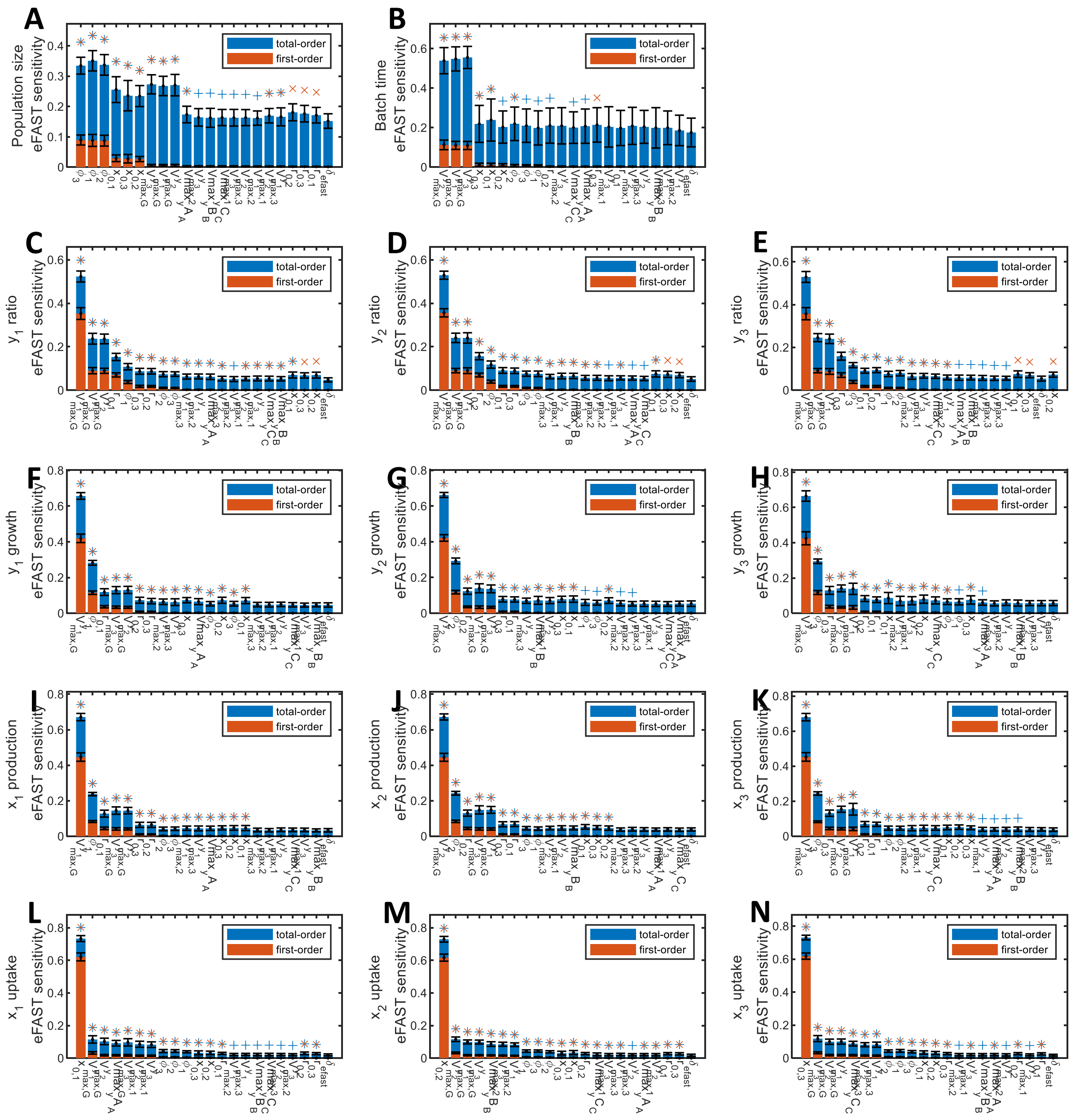

Fig. S8 Global sensitivity analysis of a three-member co-culture with multi-directional metabolite exchange and re-uptake.

The global sensitivity analysis was carried out as described in **Supplementary Note 1.** Full results are shown from each performance metric. Orange bars represent the first order sensitivities. Blue bars represent the total order sensitivities. 100 re-samplings were carried out with the mean and standard deviation in the sensitivity reported. Statical significance of the sensitivities relative to the dummy parameter $\delta efast$ was calculated using t-test with an α value of 0.01 adjusted using Bonferroni correction by the number of parameters being tested. “+”, sensitivity is significantly different ($p < 0.01$) from the dummy parameter. “x”, the total sensitivity is significantly different ($p < 0.01$) from the dummy parameter.

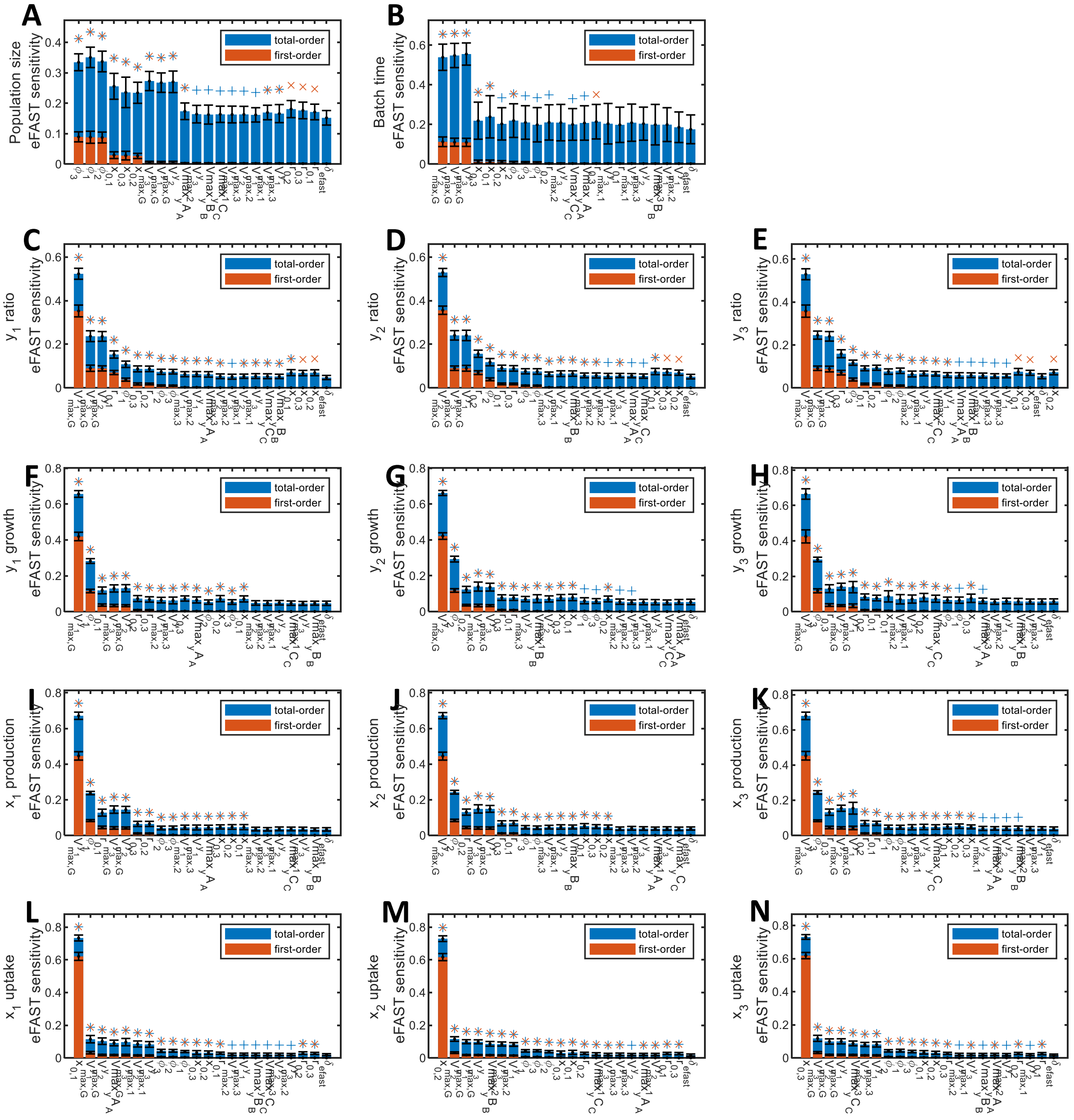

Fig. S9 Global sensitivity analysis of the division of labour system

The global sensitivity analysis was carried out as described in **Supplementary Note 1.** Full results are shown from each performance metric. Orange bars represent the first order sensitivities. Blue bars represent the total order sensitivities. 100 re-samplings were carried out with the mean and standard deviation in the sensitivity reported. Statical significance of the sensitivities relative to the dummy parameter $\delta efast$ was calculated using t-test with an α value of 0.01 adjusted using Bonferroni correction by the number of parameters being tested. "+", sensitivity is significantly different ($p < 0.01$) from the dummy parameter. "x", the total sensitivity is significantly different ($p < 0.01$) from the dummy parameter.

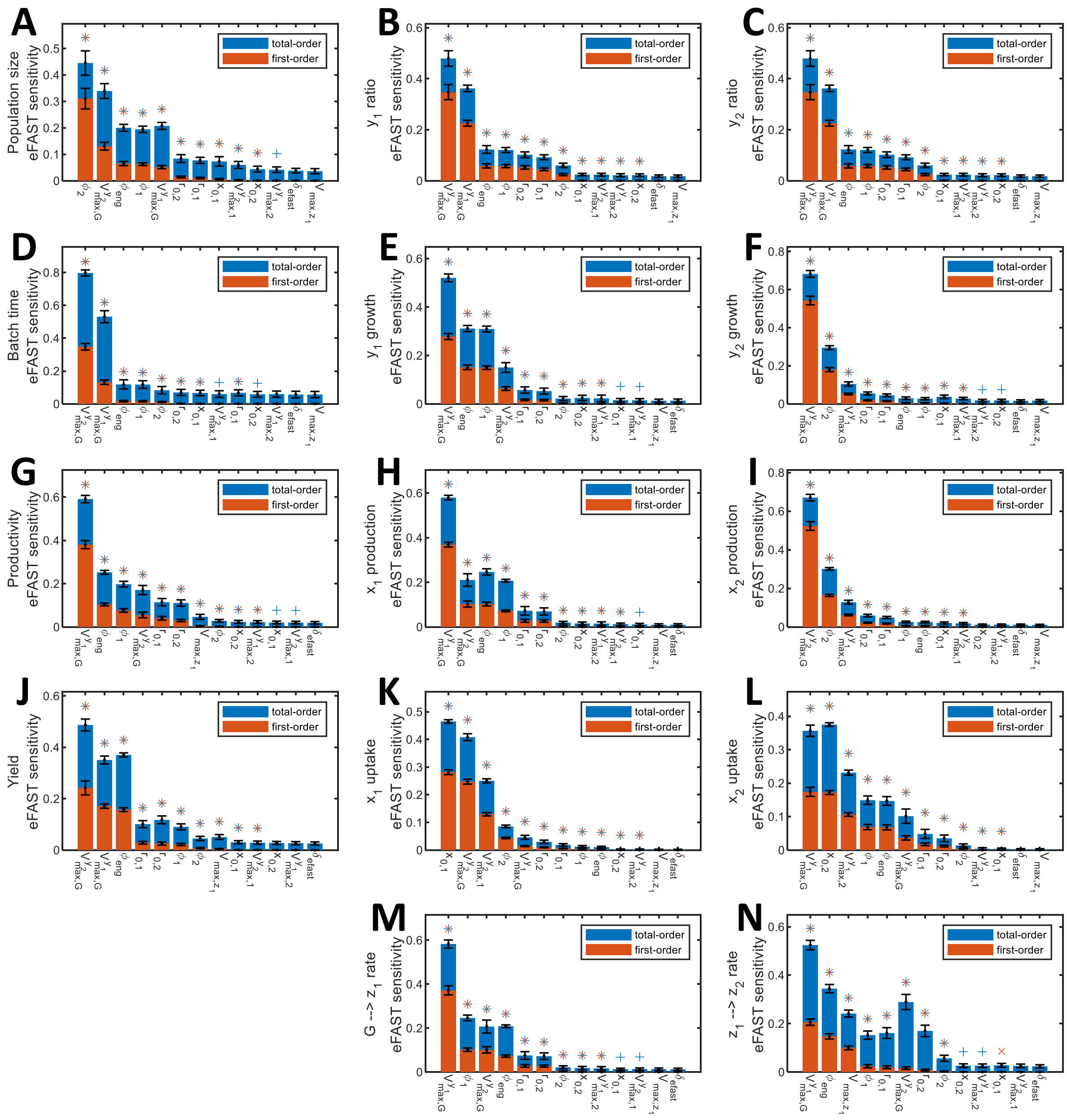

Fig. S10 Global sensitivity analysis of the division of labour system with key pathway parameters also varied.

The global sensitivity analysis was carried out as described in **Supplementary Note 1.** Full results are shown from each performance metric. Orange bars represent the first order sensitivities. Blue bars represent the total order sensitivities. 100 re-samplings were carried out with the mean and standard deviation in the sensitivity reported. Statical significance of the sensitivities relative to the dummy parameter $\delta efast$ was calculated using t-test with an α value of 0.01 adjusted using Bonferroni correction by the number of parameters being tested. "+", sensitivity is significantly different ($p < 0.01$) from the dummy parameter. "x", the total sensitivity is significantly different ($p < 0.01$) from the dummy parameter.

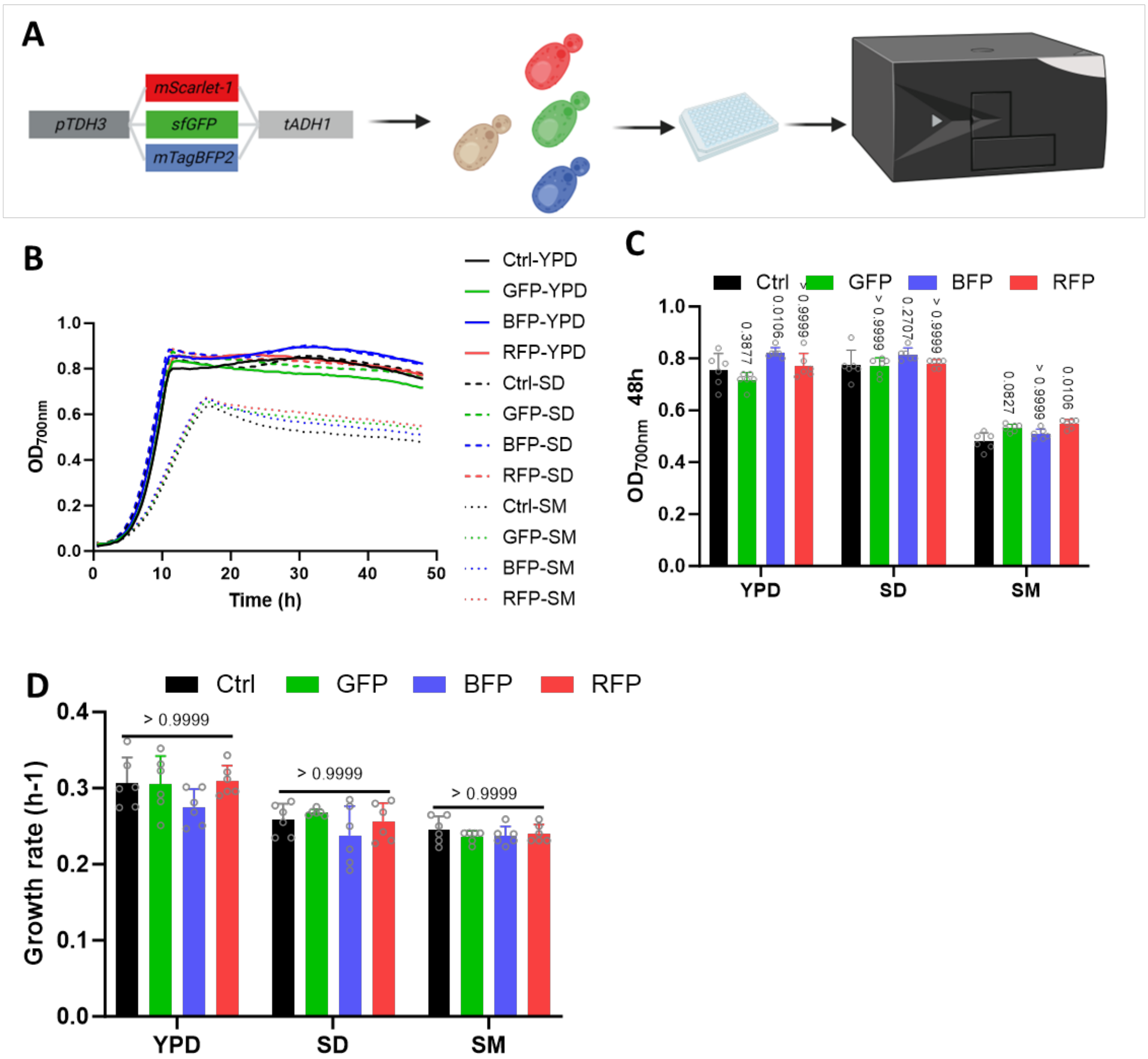

Fig. S11 Assessment of the expression of three fluorescence proteins on yeast cell growth in different medium.

A. Three fluorescence proteins (FPs) including sfGFP (GFP), mTagBFP2 (BFP), mScarlet-I (RFP) were assembled between the pTDH3 promoter and tADH1 terminator, and then integrated into the LEU2 locus. BY4741-pHLUM was used as a control (Ctrl). Four strains were tested for 48 h in different medium including YPD (yeast extract peptone dextrose), SD (synthetic complete dextrose) and SM (synthetic minimal) in a Tecan Spark plate reader. **B.** Comparison of cell growth curves, **C.** OD_{700nm} values at 48 h and **D.** growth rate (h^{-1}) of these four strains in different medium. N = six biologically independent samples, and data are presented as mean values +/- SD. Two-way ANOVA, followed by Bonferroni's multiple comparisons test with 95% confidence intervals were performed using Prism 9.5.0 (GraphPad) software, and p values were noted.

To select stable FPs for our co-culture system, we assessed changes in expression of three FPs (sfGFP, mTagBFP2, mScarlet-I) when integrated into the BY4741 genome, using a LEU2 integration vector under strong promoter pTDH3 and terminator tADH1. Three different culture mediums including YPD (yeast extract peptone dextrose), SD (synthetic complete dextrose), SM (synthetic minimal) were used to culture the FP labelled yeast cells. Cell growth of these three fluorescence labelled strains, plus the control (BY4741), were measured and compared within 48 h in different medium. In the comparative

analysis of fluorescent protein expression, specifically BFP vs. Ctrl in YPD and RFP vs. Ctrl in SM, a statistically significant result denoted by p-value = 0.0106 at 48 h was obtained. However, it is important to note that the growth rates of these four strains are not significant different among different media. Across various time points within 48 h, the majority of growth patterns between the different strains cultured in the same medium were comparable. These observations collectively indicate that the expression of GFP, BFP, and RFP had minimal discernible impact on the yeast growth trajectories within the investigated time frames and under the specified culture conditions. Due to the richness of YPD and SD media, and their lack of commercial options for alternate dropout amino acid formulations, SM was chosen for the subsequent cross-feeding co-culture experiments.

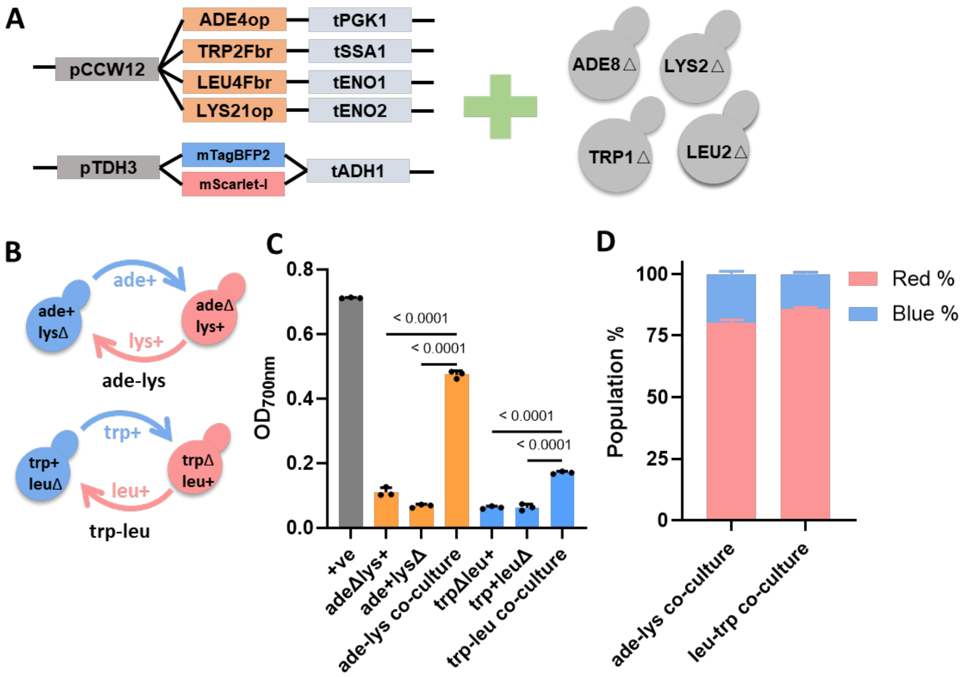

Fig. S12 Validation of reported two pairs of cross-feeding co-cultures.
A. Cassettes of four feedback resistant genes and two fluorescent proteins of mTagBFP2 and mScarlet-I, and four auxotrophic BY4741 strains. **B.** Diagram of two pairs of cross-feeding co-cultures ade-lys, trp-leu. **C.** OD_{700nm} values at 72 h of these two pairs of co-cultures and monoculture controls. **D.** Population percentages (red and blue) of each member of these two co-cultures at 72 h. +ve (positive control) was BY4741_pHLUM. N = three biologically independent samples, and data are presented as mean values +/- SD. One-way ANOVA, followed by Bonferroni's multiple comparisons test with 95% confidence intervals were performed using Prism 9.5.0 (GraphPad) software, and p values were noted.

Two reported two-member cross-feeding co-cultures of ade-lys (Shou et al.2007) and leu-trp (Muller et al. 2014) were validated first before we explored more targets that can be used for developing the co-culture toolkit. For these two pairs of two-member cross-feeding co-cultures, four feedback resistant genes that help nucleotide (ade) and amino acid (lys, leu and trp) production were assembled to a stronger promoter *pCCW12* and different terminators. Two fluorescent proteins (mTagBFP2 and mScarlet-I) were used as markers in the co-cultures. Both ade-lys and trp-leu co-cultures showed significantly higher cell growth than the monoculture controls, and red member was dominant in both co-cultures.

References

Shou, W.Y., Ram, S. & Vilar, J.M.G. Synthetic cooperation in engineered yeast populations. P Natl Acad Sci USA 104, 1877-1882 (2007).

Muller, M.J.I., Neugeboren, B.I., Nelson, D.R. & Murray, A.W. Genetic drift opposes mutualism during spatial population expansion. P Natl Acad Sci USA 111, 1037-1042 (2014).

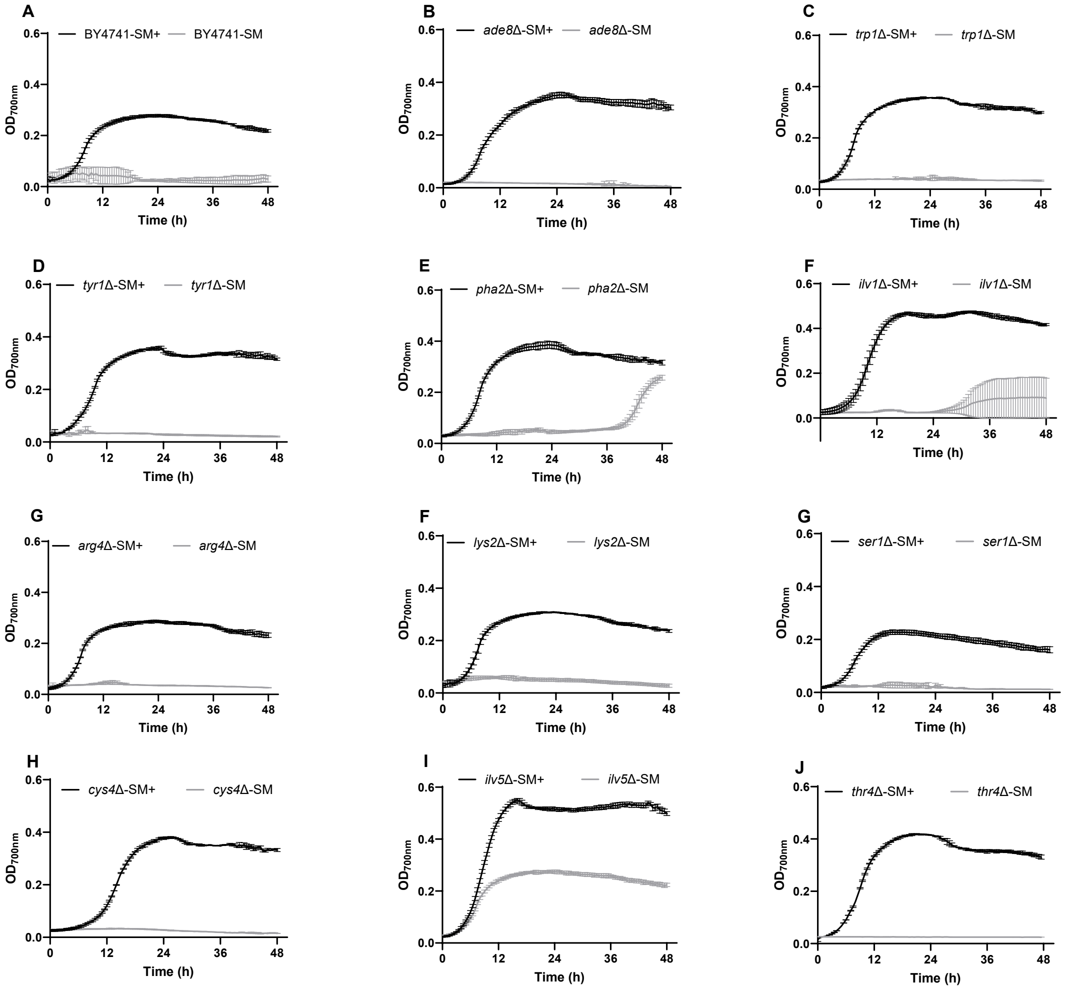

Fig. S13 Cell growth curves of auxotrophic strains with and without amino acid and nucleotide supplementation in synthetic minimal medium.

A-J. Auxotrophic strains include BY4741 (*his3Δ, leu2Δ, ura3Δ, met15Δ*), *ade8Δ, trp1Δ, tyr1Δ, pha2Δ, ilv1Δ, arg4Δ, lys2Δ, ser1Δ, cys4Δ, ilv5Δ, thr4Δ.* Strain BY4741 is auxotrophic to four different metabolites of his, leu, ura and met. Single auxotrophic strain of his, leu, ura and met can be created by transforming BY4741 with pHLUM plasmid easily, and strain *aro7Δ* is auxotrophic to both phe and tyr, were not shown here. Strain – SM, negative control; strain – SM+, positive control with supplemented metabolites. N = three biologically independent samples, and data are presented as mean values +/- SD.

All these auxotrophic strains were obtained either from the YKO library or generated by using CRISPR-cas9, followed by colony PCR and sequencing verification (**Tab. S1**). We also verified all auxotrophic strains by a cell growth assay in synthetic minimal medium with and without each cell's required amino acid or nucleotide supplemented (SM, SM+, **Tab. S2**). Most knockout strains showed expected phenotypes corresponding to their genotypes, namely, the auxotrophic strains should only be able to grow in SM+, but not in SM. However, several strains did not show strict auxotrophic phenotypes, where they either started to grow at around 30 h, such as *pha2Δ, ilv1Δ*, or showed reduced cell growth in SM, such as *ilv5Δ*.

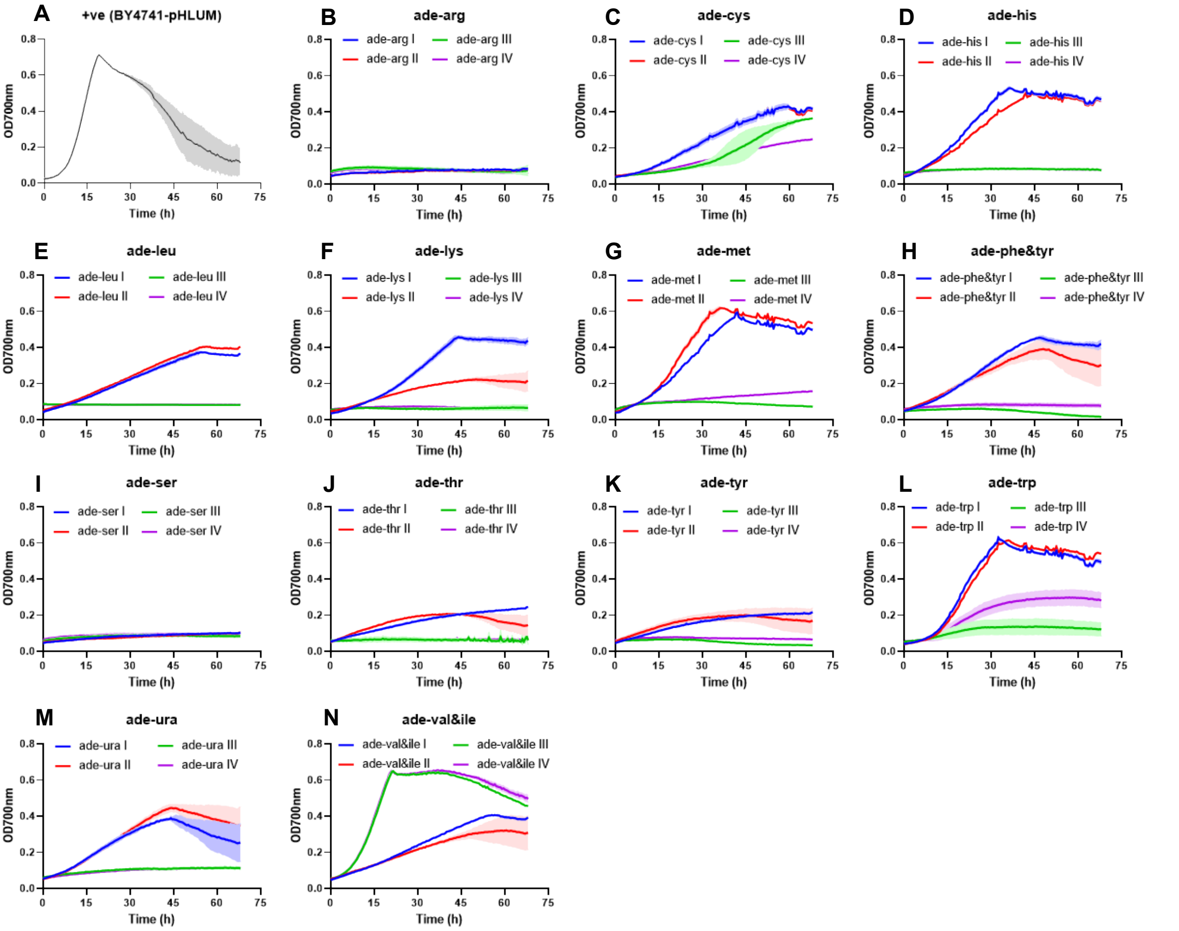

Fig. S14 Time courses of total OD values of adenine-exchanged metabolite I-IV co-cultures in synthetic minimal medium

A. +ve, positive control using BY4741-pHLUM - in our opinion, the observed drop in OD is a technical issue linked to cell sedimentation observed at high cell densities in small volumes; **B.** ade-arg I, II, III, IV; **C**. ade-cys I, II, III, IV; **D.** ade-his I, II, III, IV; **E**. ade-leu I, II, III, IV; **F**. ade-lys I, II, III, IV; **G**. ade-met I, II, III, IV; **H**. ade-phe&tyr I, II, III, IV; **I**. ade-ser I, II, III, IV; **J.** ade-thr I, II, III, IV; **K**. ade-tyr I, II, III, IV; **L**. ade-trp I, II, III, IV; **M**. ade-ura I, II, III, IV; **N**. ade-val&ile I, II, III, IV. N = three biologically independent samples, and data are presented as mean values +/- SD.

Fig. S14 is a complementary to main **Fig. 2.**

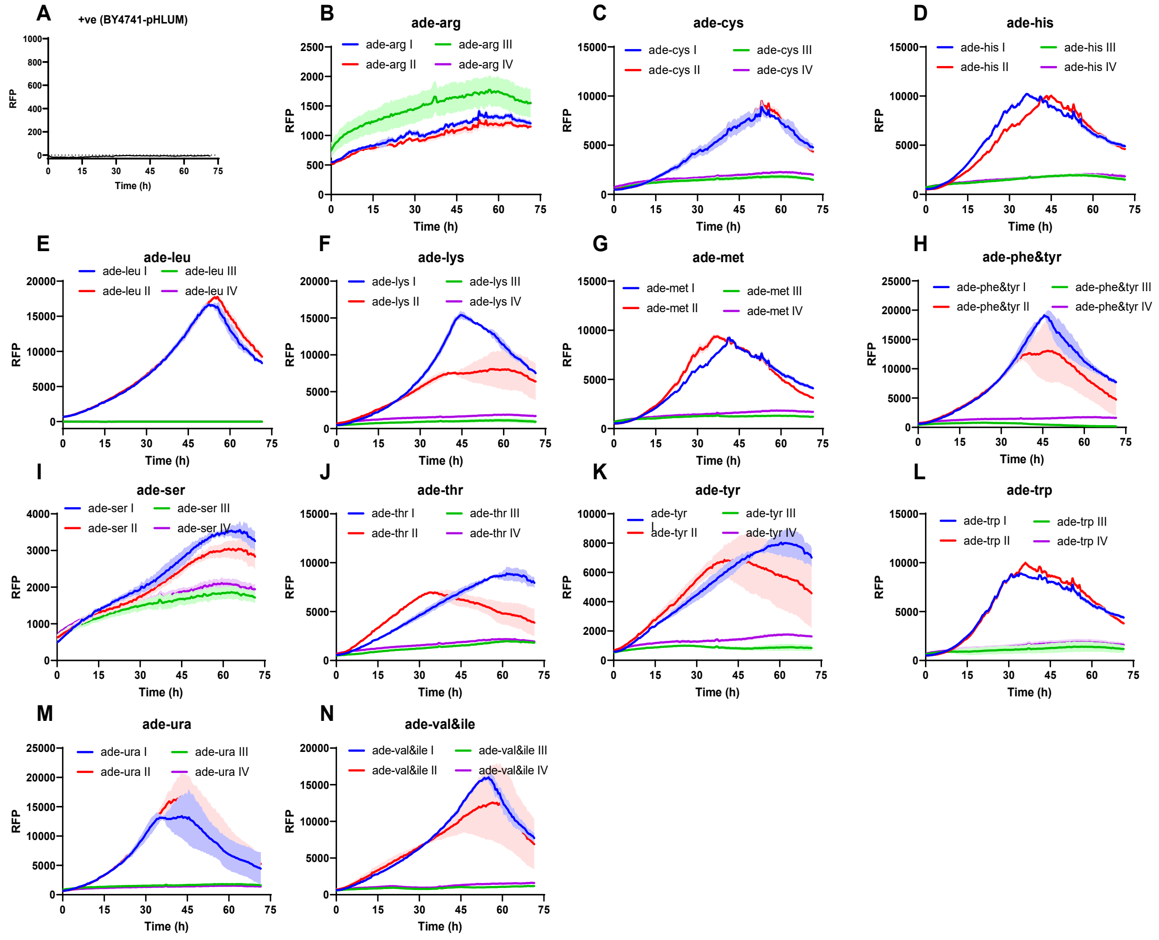

Fig. S15 Time courses of red fluorescent intensities of adenine-exchanged metabolite I-IV co-cultures in synthetic minimal medium

A. +ve, positive control using BY4741-pHLUM; **B.** ade-arg I, II, III, IV; **C**. ade-cys I, II, III, IV; **D.** ade-his I, II, III, IV; **E**. ade-leu I, II, III, IV; **F**. ade-lys I, II, III, IV; **G**. ade-met I, II, III, IV; **H**. ade-phe&tyr I, II, III, IV; **I**. ade-ser I, II, III, IV; **J.** ade-thr I, II, III, IV; **K**. ade-tyr I, II, III, IV; **L**. ade-trp I, II, III, IV; **M**. ade-ura I, II, III, IV; **N**. ade-val&ile I, II, III, IV. N = three biologically independent samples, and data are presented as mean values +/- SD.

Fig. S15 is a complementary to main **Fig. 2.**

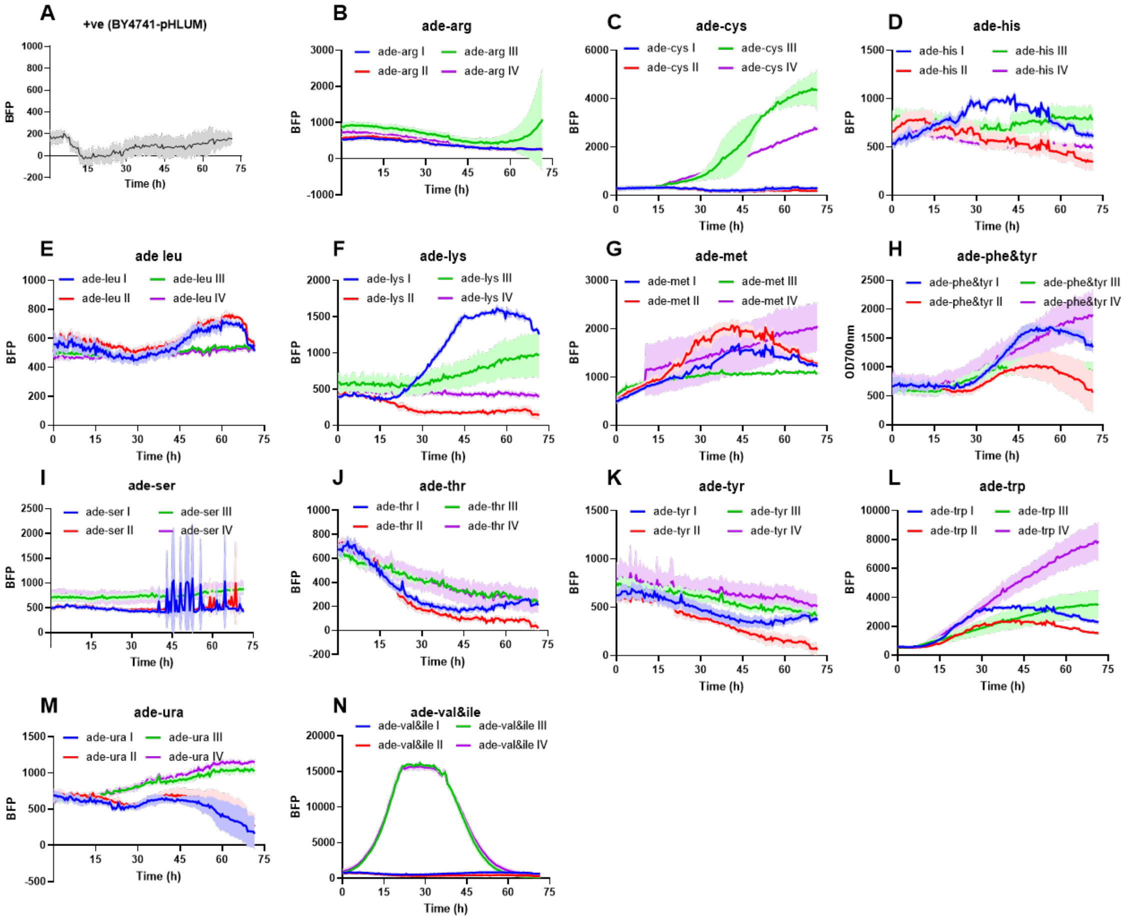

Fig. S16 Time courses of blue fluorescent intensities of adenine- exchanged metabolite I-IV co-cultures in synthetic minimal medium

A. +ve, positive control using BY4741-pHLUM; **B.** ade-arg I, II, III, IV; **C**. ade-cys I, II, III, IV; **D.** ade-his I, II, III, IV; **E**. ade-leu I, II, III, IV; **F**. ade-lys I, II, III, IV; **G**. ade-met I, II, III, IV; **H**. ade-phe&tyr I, II, III, IV; **I**. ade-ser I, II, III, IV; **J.** ade-thr I, II, III, IV; **K**. ade-tyr I, II, III, IV; **L**. ade-trp I, II, III, IV; **M**. ade-ura I, II, III, IV; **N**. ade-val&ile I, II, III, IV. N = three biologically independent samples, and data are presented as mean values +/- SD.

Fig. S16 is a complementary to main **Fig. 2.**

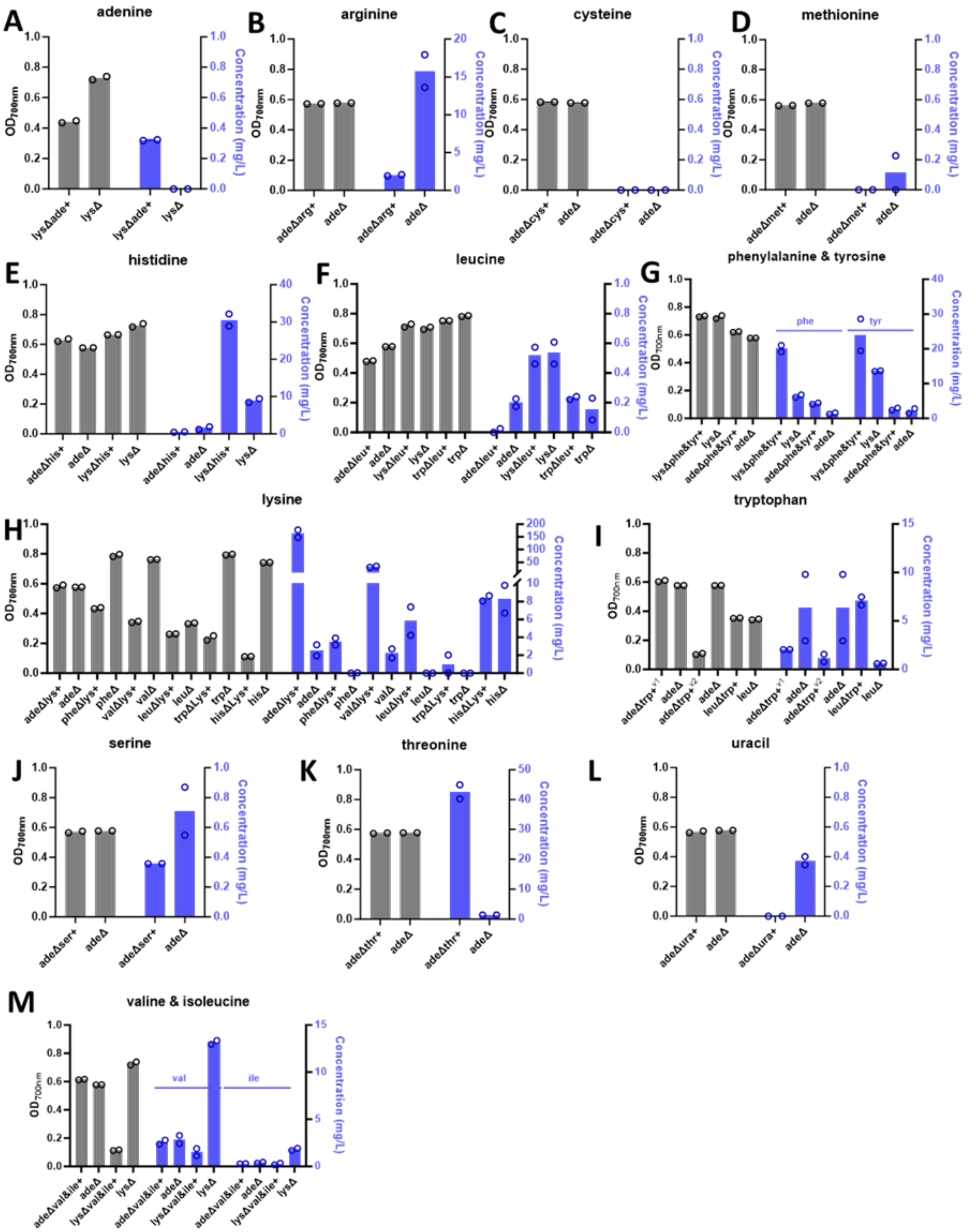
A
adenine
OD700nm
Concentration (mg/L)
lysΔade+
lysΔ
B
arginine
adeΔarg+
adeΔ
C
cysteine
adeΔcys+
D
methionine
adeΔmet+
E
histidine
adeΔhis+
lysΔhis+
F
leucine
adeΔleu+
lysΔleu+
trpΔleu+
trpΔ
G
phenylalanine & tyrosine
phe
tyr
lysΔphe&tyr+
adeΔphe&tyr+
H
lysine
adeΔlys+
pheΔlys+
pheΔ
valΔlys+
valΔ
leuΔlys+
leuΔ
trpΔLys+
hisΔLys+
hisΔ
I
tryptophan
adeΔtrp+ v1
adeΔtrp+ v2
leuΔtrp+
J
serine
adeΔser+
K
threonine
adeΔthr+
L
uracil
adeΔura+
M
valine & isoleucine
val
ile
adeΔval&ile+
lysΔval&ile+

Fig. S17 Comparing cell growth and secretion of exchanged metabolites in auxotrophic monocultures that contain an extra-strong or native promoter for exchanged metabolite synthesis. A-M. Cell growth at OD_{700nm} and secretion of 15 distinct metabolites were measured and compared at 23 h. These exchanged metabolites encompassed adenine, arginine, cysteine, methionine, histidine, leucine, phenylalanine, tyrosine, lysine, tryptophan, serine, threonine, uracil, valine and isoleucine. "+", gene expression under extra strong promoter pCCW12; "Δ", gene knockout, auxotrophic; grey bar, OD_{700nm} at 23 h; blue bar, the concentration of exchanged metabolites, mg/L. The error bars presented in the figures correspond to the standard deviation. N = two biologically independent samples, and data are presented as mean values.

To obtain insight into the physiological parameters of the individual auxotrophic monocultures with and without the extra expression of exchanged metabolites, which served as the basis for the subsequent co-culture experimental design. We cultured the monocultures in synthetic minimal medium at 30 °C, 250 rpm, supplemented with the necessary auxotrophic metabolites corresponding to each strain. Then, we sampled the monocultures at 23 h (during or immediately prior to stationary phase) and quantified both cell growth (OD_{700nm}) and exchanged metabolites (by LC-MS, the raw data can be found in Source Data). These monocultures included 15 exchanged metabolites and could be used for the following co-cultures: ade-arg, ade-cys, ade-his, ade-leu, ade-lys, ade-met, ade-phe&tyr, ade-ser, ade-thr, ade-trp, ade-ura, ade-val, ade-trp, leu-trp, phe-lys, val-lys, leu-lys, trp-lys and his-lys.

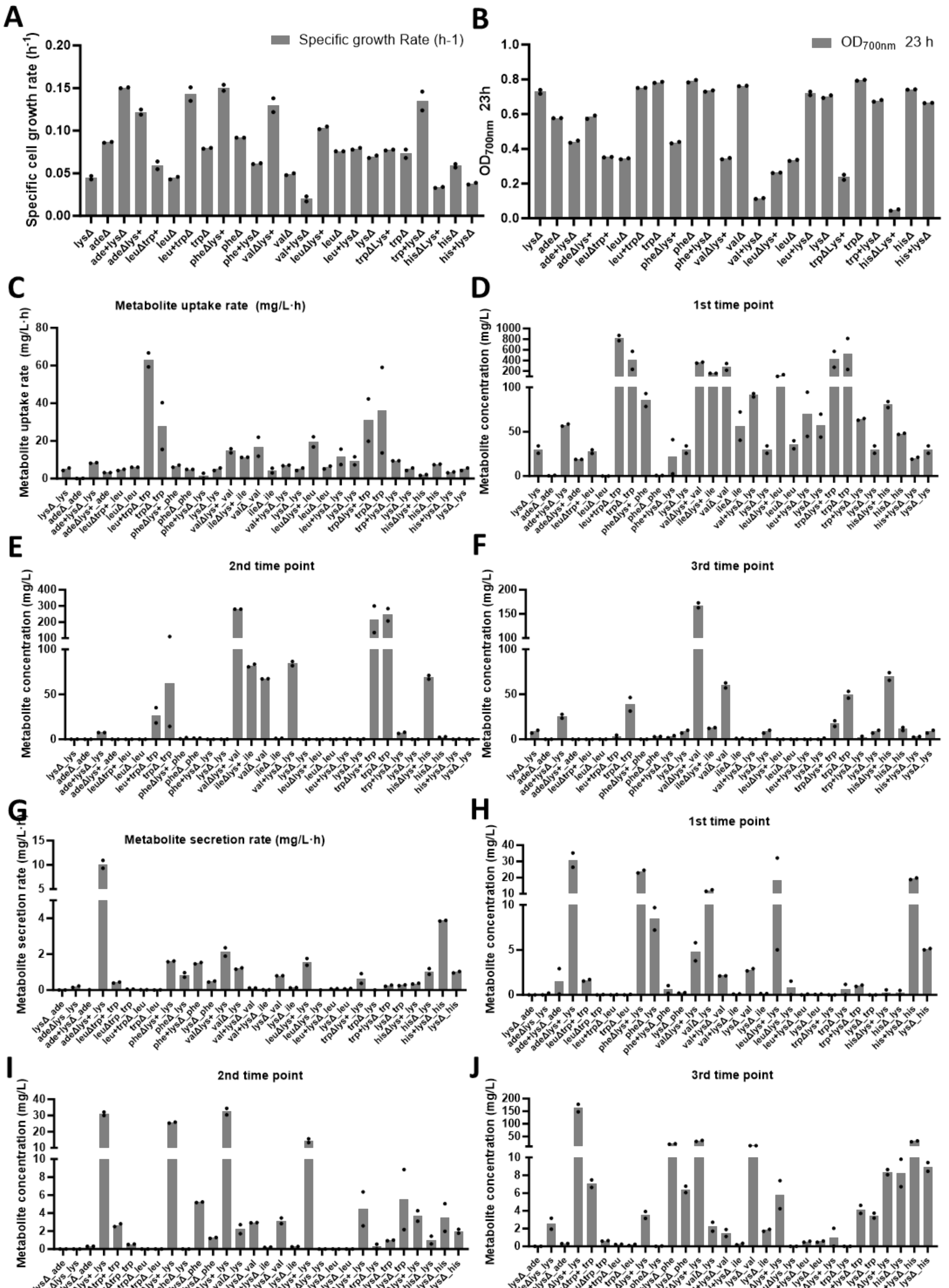

A
Specific growth Rate (h-1)
Specific cell growth rate (h-1)
B
OD700nm 23 h
OD700nm 23h
C
Metabolite uptake rate (mg/L·h)
Metabolite uptake rate (mg/L·h)
D
1st time point
Metabolite concentration (mg/L)
E
2nd time point
Metabolite concentration (mg/L)
F
3rd time point
Metabolite concentration (mg/L)
G
Metabolite secretion rate (mg/L·h)
Metabolite secretion rate (mg/L·h)
H
1st time point
Metabolite concentration (mg/L)
I
2nd time point
Metabolite concentration (mg/L)
J
3rd time point
Metabolite concentration (mg/L)

Fig. S18 Cell growth, exchanged metabolite uptake and secretion of auxotrophic monocultures that contain strong and native promoter for exchanged metabolite synthesis.

A. Specific growth rate (h^{-1}) of auxotrophic monocultures; **B.** OD_{700nm} value at 23 h of auxotrophic monocultures; **C.** Uptake rate of supplemented metabolites (mg/L h); **D-F.** Remaining concentration of supplemented metabolites (mg/L) for auxotrophic monocultures at the 1st, 2nd and 3rd time points. **G.** Secretion rate of target metabolites (mg/L h); **H-J.** The concentration of secreted target metabolites (mg/L) at the 1st, 2nd and 3rd time points. N = two biologically independent samples, and data are presented as mean values. The three time points were 10 h, 16 h and 23 h for most strains, and different for some strains that grew slowly. Detailed information can be found in Source Data.

Fig.S18 is a complementary to **Fig. S17.**

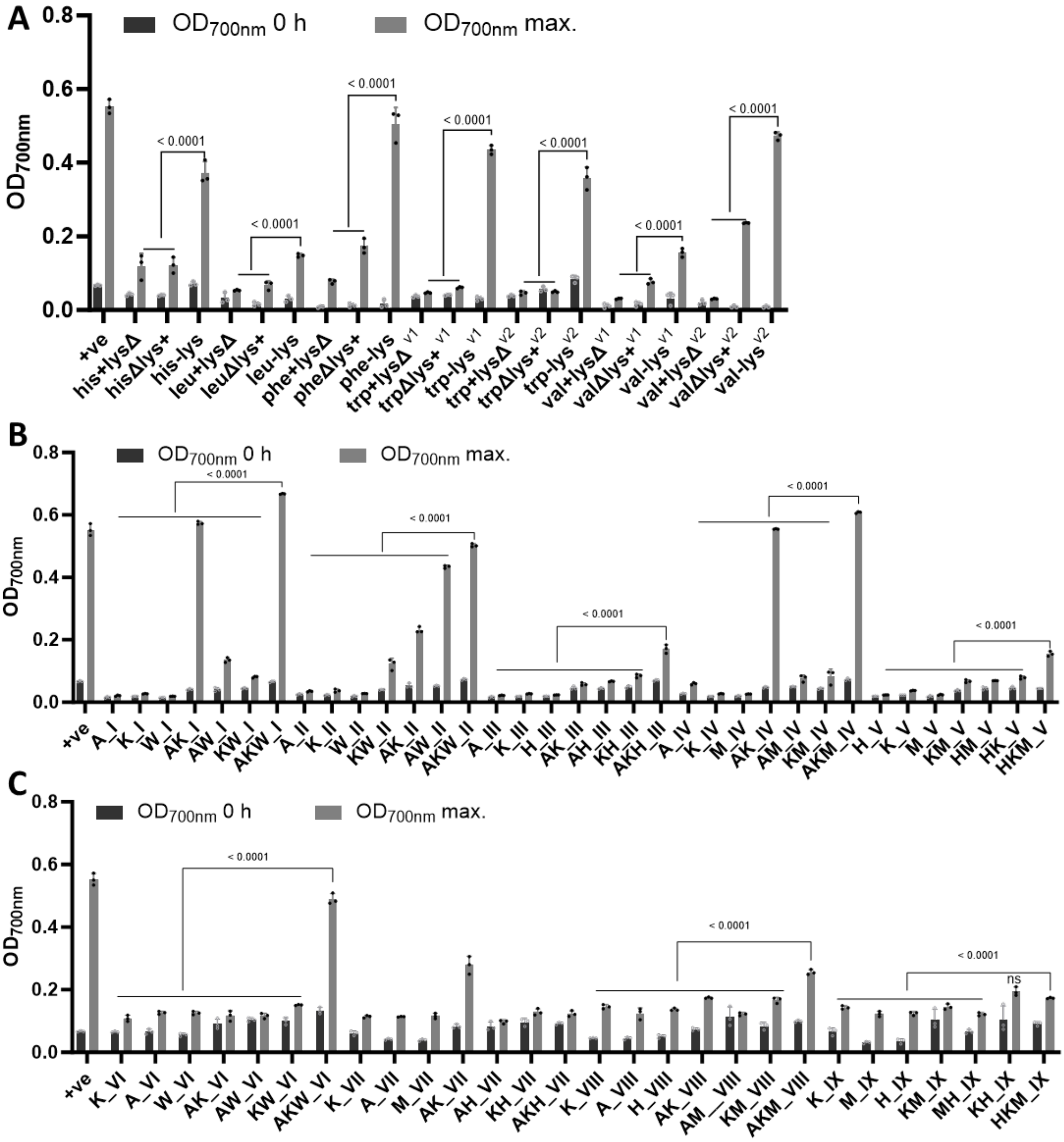

Fig. S19 Initial and maximal OD_{700nm} values of two-member and three-member co-cultures and their controls of monocultures and two-member co-cultures within 72 h.

A. Initial (0 h) and maximal OD_{700nm} values of the two-member co-cultures and their controls of auxotrophic monocultures within 72 h; **B-C.** Initial (0 h) and maximal OD_{700nm} values of the three-member co-cultures and their controls of monocultures and two-member co-cultures within 72 h. N = three biologically independent samples, and data are presented as mean values +/- SD. Two-way ANOVA, followed by Bonferroni's multiple comparisons test with 95% confidence intervals were performed using Prism 9.5.0 (GraphPad) software, and p values were noted.

Fig.S19 is a complementary to main **Fig. 3.**

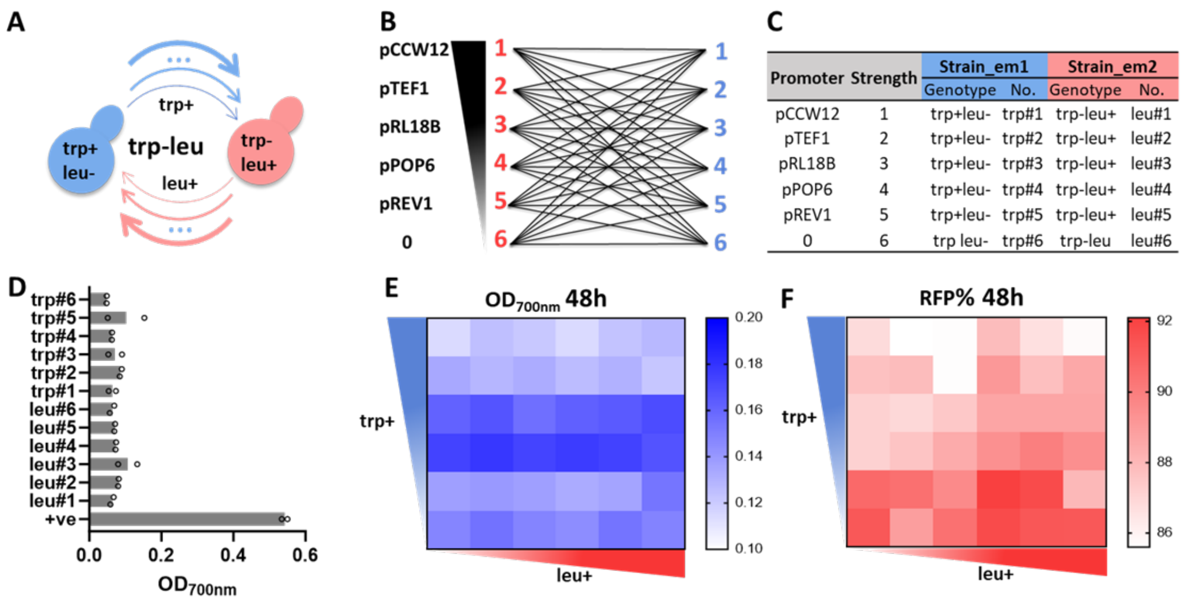

Fig. S20 Promoter engineering controlled cell growth and population size in tryptophan-leucine two-member cross-feeding co-cultures.

A. Diagram of tryptophan-leucine (trp-leu) two-member cross-feeding co-cultures, blue member is overexpressing *TRP2Fbr* and auxotrophic to leu, red member is overexpressing *LEU4Fbr* and auxotrophic to trp. **B.** The orthogonal combinations of six promoters with different strengths from strong to weak (1-6). *TRP2Fbr, LEU4Fbr* were driven by these six promoters in blue and red members, respectively. Then, these 6x blue and 6x red members were orthogonally combined to form 36 (6x6) different two-member co-cultures. **C.** Strain table for the combinations of trp-leu two-member cross-feeding co-cultures. Abbreviations represent target gene and the promoter strength, e.g., the blue strain trp+leu- overexpressing *TRP2Fbr* under stronger promoter *pCCW12*, is encoded as trp#1. **D.** OD_{700nm} at 48 h of monocultures in synthetic minimal medium, with negative and positive controls (+ve, using BY4741-pHLUM). **E-F**. Heatmap of both OD_{700nm} values and red population percentages at 48 h for the 6x6 trp-leu orthogonal two-member co-cultures. In this co-culture setup, the initial ratio was 1:1, and the initial cell density was OD_{700nm} 0.078 for each member. X-axis from left to right means the promoter strength of leu+ (*LEU4Fbr*) from weak (6) to strong (1), y-axis from down to up means the promoter strength of trp+ (*TRP2Fbr*) from weak (6) to strong (1). N = two biologically independent samples, and data are presented as mean values +/- SD.

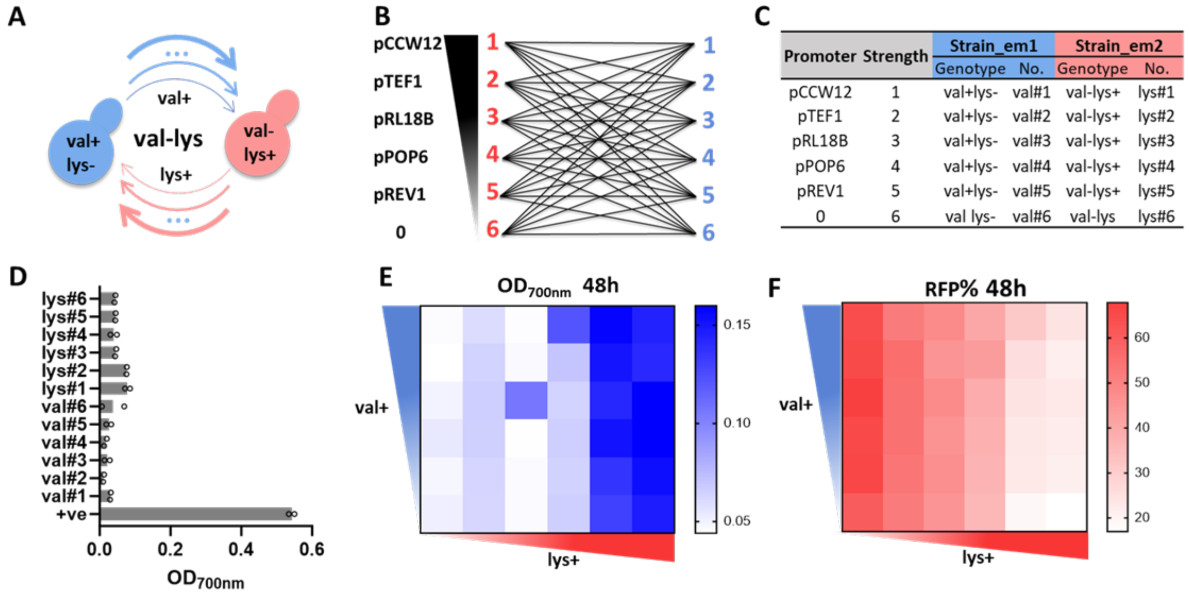

Fig. S21 Promoter engineering controlled cell growth and population size in valine - lysine two-member cross-feeding co-cultures.

A. Diagram of valine-lysine (val-lys) two-member cross-feeding co-cultures, blue member is overexpressing *ILV6G89D* and auxotrophic to lys, red member is overexpressing *LYS21op* and auxotrophic to val. **B.** The orthogonal combinations of six promoters with different strengths from strong to weak (1-6). *ILV6G89D, LYS21op* were driven by these six promoters in blue and red members, respectively. Then, these 6x blue and 6x red members were orthogonally combined to form 36 (6x6) different two-member co-cultures. **C.** Strain table for the combinations of val-lys two-member cross-feeding co-cultures. Abbreviations represent target gene and promoter strength, such as blue strain val+lys- overexpressing *ILV6G89D* under stronger promoter *pCCW12*, is encoded as val#1. **D.** OD_{700nm} at 48 h of monocultures in synthetic minimal medium, with negative and positive controls (+ve, using BY4741-pHLUM). **E-F**. Heatmap of both OD_{700nm} values and red population percentages at 48 h for the 6x6 val-lys orthogonal two-member co-cultures. In this co-culture setup, the initial ratio was 1:1, and the initial cell density was OD_{700nm} 0.078 for each member. X-axis from left to right means the promoter strength of val+ (*ILV6G89D*) from weak (6) to strong (1), y-axis from down to up means the promoter strength of lys+ (*LYS21op*) from weak (6) to strong (1). N = two biologically independent samples, and data are presented as mean values +/- SD.

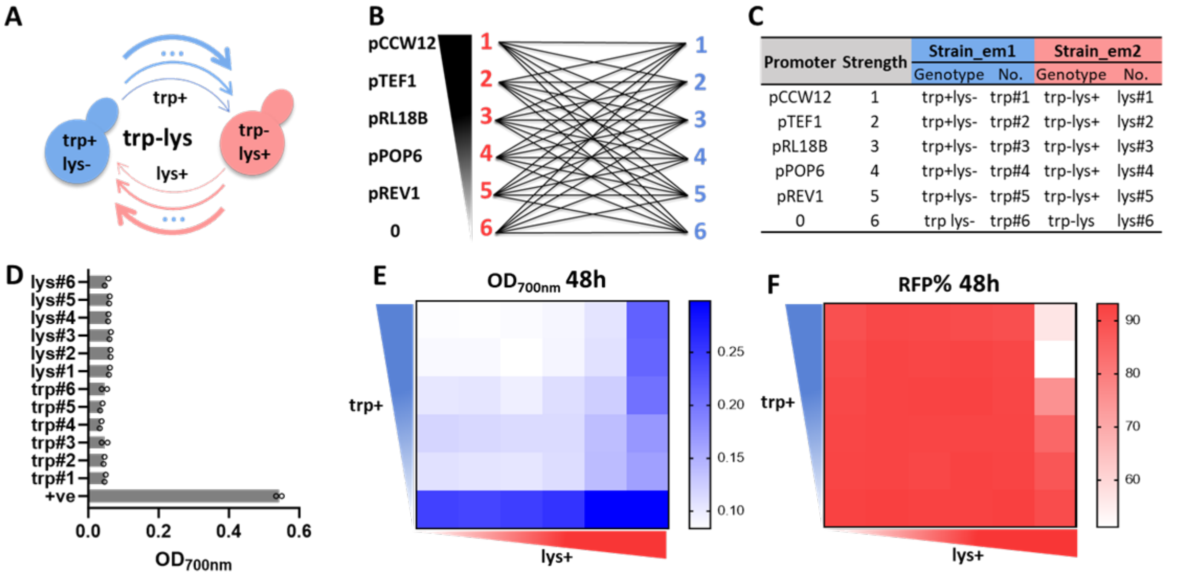

Fig. S22 Promoter engineering controlled cell growth and population size in tryptophan - lysine two-member cross-feeding co-cultures.

A. Diagram of tryptophan-lysine (trp-lys) two-member cross-feeding co-cultures, blue member is overexpressing *TRP2Fbr* and auxotrophic to lys, red member is overexpressing *LYS21op* and auxotrophic to trp. **B.** The orthogonal combinations of six promoters with different strengths from strong to weak (1-6). *TRP2Fbr, LYS21op* were driven by these six promoters in blue and red members, respectively. Then, these 6x blue and 6x red members were orthogonally combined to form 36 (6x6) different two-member co-cultures. **C.** Strain table for the combinations of trp-lys two-member cross-feeding co-cultures. Abbreviations represent target gene and promoter strength, such as blue strain trp+lys- overexpressing *TRP2Fbr* under stronger promoter *pCCW12*, is encoded as trp#1. **D.** OD_{700nm} at 48 h of monocultures in synthetic minimal medium, with negative and positive controls (+ve, using BY4741-pHLUM). **E-F**. Heatmap of both OD_{700nm} values and red population percentages at 48 h for the 6x6 trp-lys orthogonal two-member co-cultures. In this co-culture setup, the initial ratio was 1:1, and the initial cell density was OD_{700nm} 0.078 for each member. X-axis from left to right means the promoter strength of trp+ (*TRP2Fbr*) from weak (6) to strong (1), y-axis from down to up means the promoter strength of lys+ (*LYS21op*) from weak (6) to strong (1). N = two biologically independent samples, and data are presented as mean values +/- SD.

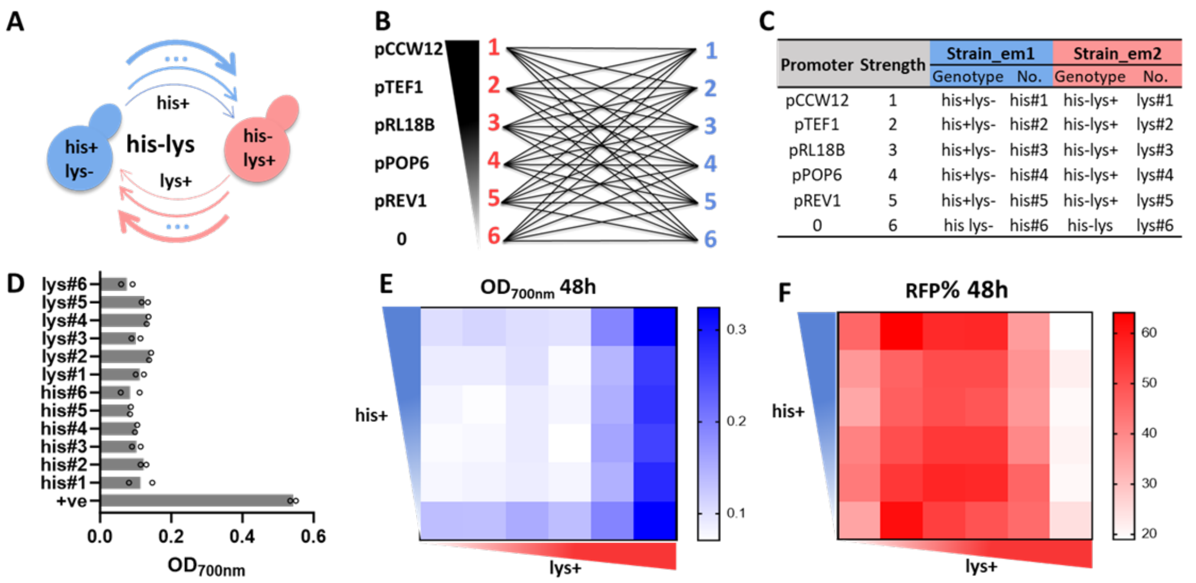

Fig. S23 Promoter engineering controlled cell growth and population size in histidine -lysine two-member cross-feeding co-cultures.

A. Diagram of histidine-lysine (his-lys) two-member cross-feeding co-cultures, blue member is overexpressing *HIS1* and auxotrophic to lys, red member is overexpressing *LYS21op* and auxotrophic to his. **B.** The orthogonal combinations of six promoters with different strengths from strong to weak (1-6). *HIS1, LYS21op* were driven by these six promoters in blue and red members, respectively. Then, these 6x blue and 6x red members were orthogonally combined to form 36 (6x6) different two-member co-cultures. **C.** Strain table for the combinations of his-lys two-member cross-feeding co-cultures. Abbreviations represent target gene and promoter strength, such as blue strain his+lys- overexpressing *HIS1* under stronger promoter *pCCW12*, is encoded as his#1. **D.** OD_{700nm} at 48 h of monocultures in synthetic minimal medium, with negative and positive controls (+ve, using BY4741-pHLUM). **E-F**. Heatmap of both OD_{700nm} values and red population percentages at 48 h for the 6x6 his-lys orthogonal two-member co-cultures. In this co-culture setup, the initial ratio was 1:1, and the initial cell density was OD_{700nm} 0.078 for each member. X-axis from left to right means the promoter strength of his+ (*HIS1*) from weak (6) to strong (1), y-axis from down to up means the promoter strength of lys+ (*LYS21op*) from weak (6) to strong (1). N = two biologically independent samples, and data are presented as mean values +/- SD.

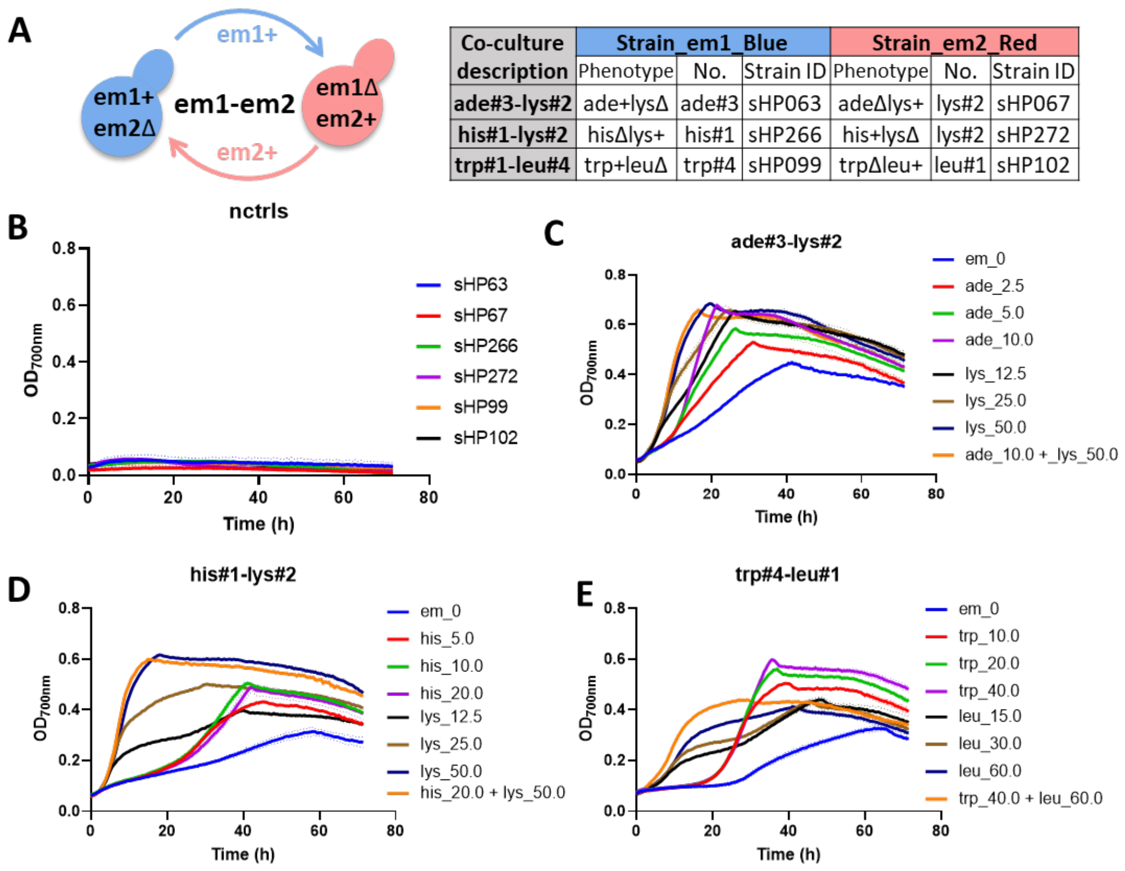

Co-culture description	Strain_em1_Blue			Strain_em2_Red		
	Phenotype	No.	Strain ID	Phenotype	No.	Strain ID
ade#3-lys#2	ade+lysΔ	ade#3	sHP063	adeΔlys+	lys#2	sHP067
his#1-lys#2	hisΔlys+	his#1	sHP266	his+lysΔ	lys#2	sHP272
trp#1-leu#4	trp+leuΔ	trp#4	sHP099	trpΔleu+	leu#1	sHP102

Fig. S24 Effects of metabolite supplementation on cell growth of three pairs of two-member co-cultures.

A. Diagram of two-member cross-feeding co-culture and strain combination table; **B.** Cell growth of monocultures as negative controls; **C-E.** Cell growth curves of three pairs of co-cultures of ade3-lys2, his1-lys2, trp4-leu1 in 72 h with metabolite supplementations, such as ade_2.5 means the final medium contains 2.5 mg/L ade supplementation. N = three biologically independent samples, and data are presented as mean values +/- SD. We chose these three pairs of two-member co-cultures as examples because they could grow reasonably in synthetic minimal medium, and each member could maintain some percentages in the co-cultures. This could be used as a baseline for the following adjustment on the cell growth and population percentages by metabolite supplementations.

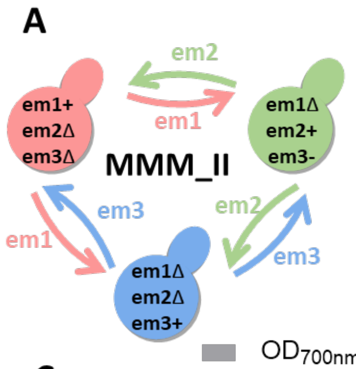

B

Three-member co-culture		Strain_em1_Red		Strain_em2_Green		Strain_em3_Blue	
description	No.	phenotype	No.	phenotype	No.	phenotype	No.
ade-lys-trp	AKW_VI	adeΔlys+trpΔ	K_VI	ade+lysΔtrpΔ	A_VI	adeΔlysΔtrp+	W_VI
ade-lys-his	AKH_VII	adeΔlys+hisΔ	K_VII	ade+lysΔhisΔ	A_VII	adeΔlysΔhis+	H_VII
ade-lys-met	AKM_VIII	adeΔlys+metΔ	K_VIII	ade+lysΔmetΔ	A_VIII	adeΔlysΔmet+	M_VIII
lys-his-met	HKM_IX	lys+hisΔmetΔ	K_IX	lysΔhisΔmet+	M_IX	lysΔhis+metΔ	H_IX

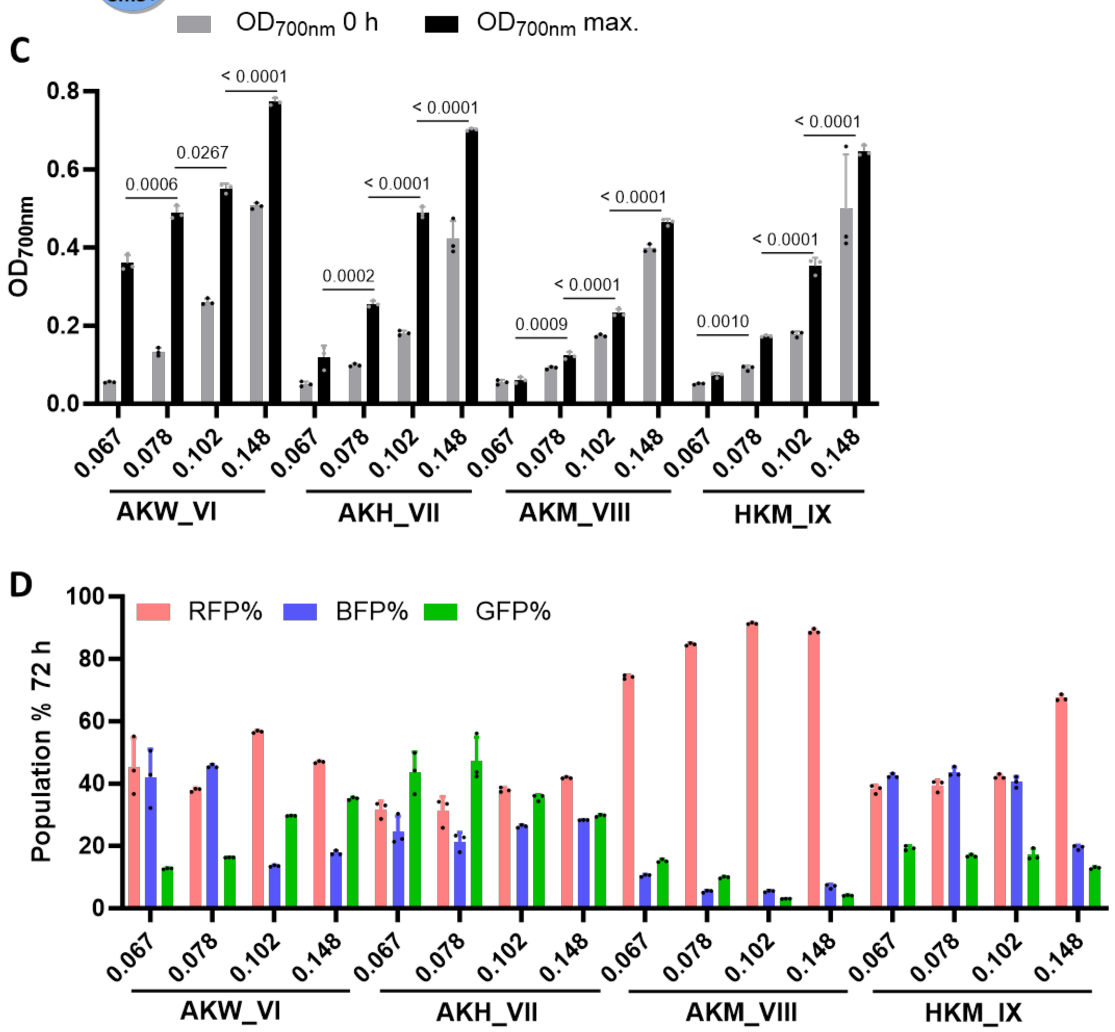

Fig. S25 Effects of initial cell densities on cell growth and population percentages of three-member co-cultures via two-way communication.

A. Diagram of three-member co-cultures via two-way communication, each member is overexpressing one gene for essential metabolite, and auxotrophic to another two essential different metabolites. **B.** Strain combination table of four pairs of three-member co-cultures. **C.** Initial and maximal OD_{700nm} values of four pairs of three-member co-cultures within 72 h under different initial cell densities. **D.** Population percentages of four pairs of three-member co-cultures at 72 h under different initial cell densities. The initial cell densities for each member were OD_{700nm} 0.067, 0.078, 0.102, 0.148 from Tecan plate reader, which equals to OD_{600nm} 0.2, 0.4, 0.8, 1.6 from the spectrophotometer, and co-culture OD values were measured by Tecan Spark plate reader. The initial ratio of each member was 1:1:1 for these four co-cultures. N = three biologically independent samples, and data are presented as mean values +/- SD. Two-way ANOVA, followed by Bonferroni's multiple comparisons test with 95% confidence intervals were performed using Prism 9.5.0 (GraphPad) software, and p values were noted.

A

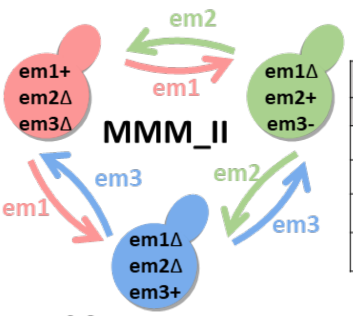

B

Three-member co-culture		Strain_em1_Red		Strain_em2_Green		Strain_em3_Blue	
description	No.	phenotype	No.	phenotype	No.	phenotype	No.
ade-lys-trp	AKW_VI	adeΔlys+trpΔ	K_VI	ade+lysΔtrpΔ	A_VI	adeΔlysΔtrp+	W_VI
ade-lys-his	AKH_VII	adeΔlys+hisΔ	K_VII	ade+lysΔhisΔ	A_VII	adeΔlysΔhis+	H_VII
ade-lys-met	AKM_VIII	adeΔlys+metΔ	K_VIII	ade+lysΔmetΔ	A_VIII	adeΔlysΔmet+	M_VIII
lys-his-met	HKM_IX	lys+hisΔmetΔ	K_IX	lysΔhisΔmet+	M_IX	lysΔhis+metΔ	H_IX

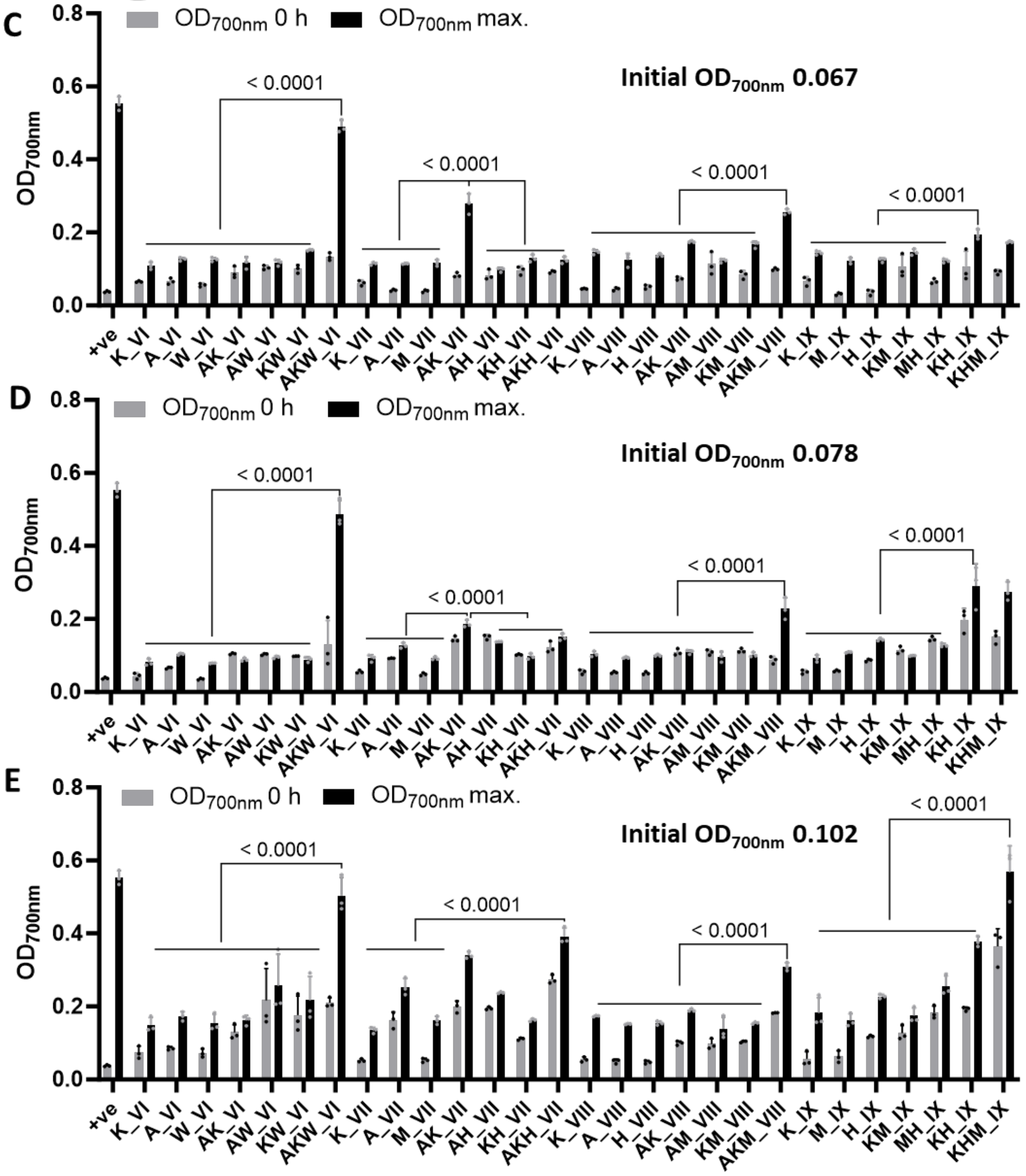

Fig. S26 Effects of initial cell densities on cell growth of three-member co-cultures via two-way communication and their negative controls.

A. Diagram of three-member co-cultures via two-way communication, each member is overexpressing one gene for essential metabolite, and auxotrophic to another two essential different metabolites. **B.** Strain combination table of four pairs of three-member co-cultures. **C-E.** The OD_{700nm} values were compared at 0 h and max. value within 72 h for four pairs of three-member co-cultures and their corresponding monoculture controls, as well as two-member co-cultures. The initial cell density was set as OD_{700nm} 0.067, OD_{700nm} 0.078 and OD_{700nm} 0.102, respectively for each member. Both initial cell densities and co-culture growth were in Tecan Spark plate reader scale. The initial ratio of each member was 1:1:1 for these four co-cultures. N = three biologically independent samples, and data are presented as mean values +/- SD. Two-way ANOVA, followed by Bonferroni's multiple comparisons test with 95% confidence intervals were performed using Prism 9.5.0 (GraphPad) software, and p values were noted. Takeaways include: i) Higher initial cell density allowed a higher co-culture growth, ii) Co-culture potential from high to low: AKW, AKM, KHM, AKH, iii) some two-member combinations showed cell growth under high initial cell density.

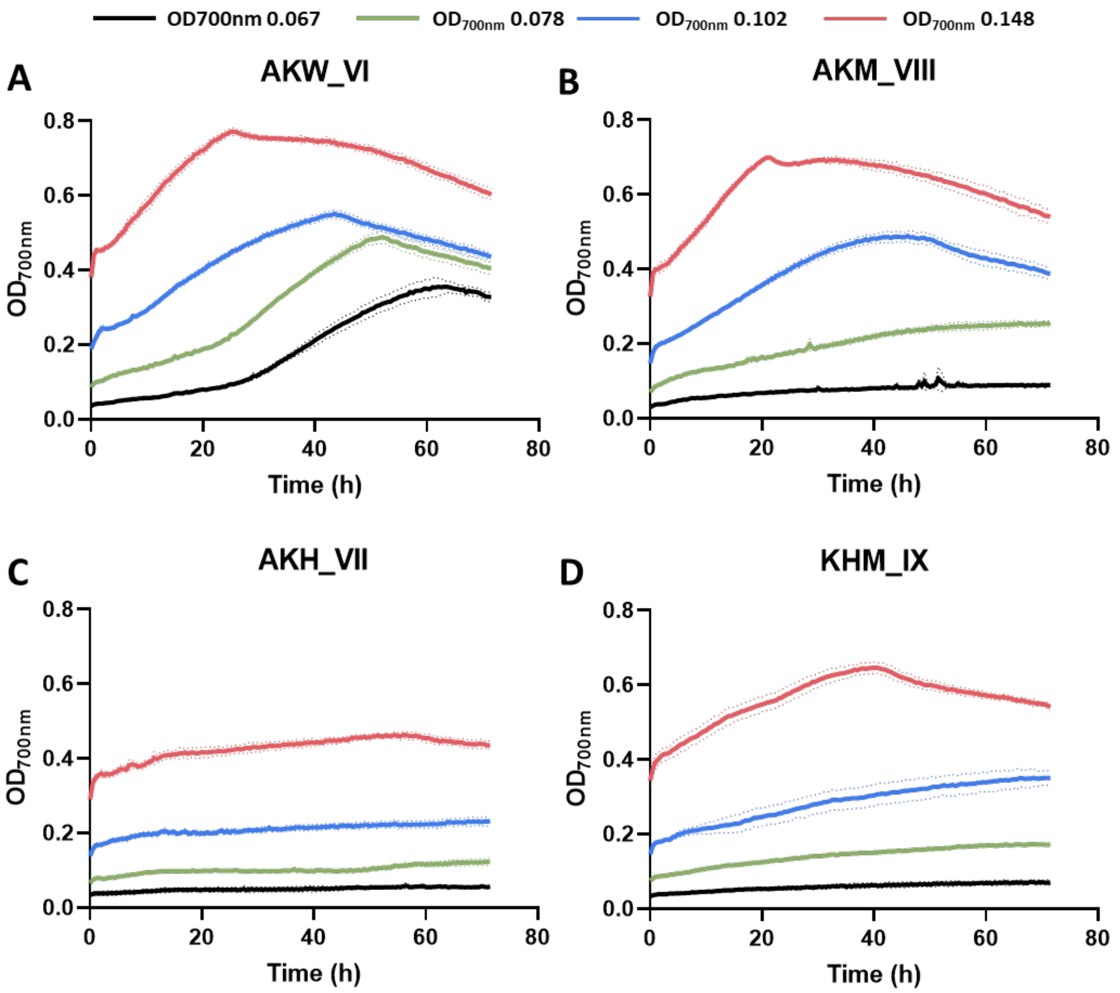

Fig. S27 Time courses of cell growth of four pairs of three-member co-cultures via two-way communication under different initial cell densities.

A-D. The growth curves of four pairs of three-member co-cultures, operating through two-way communication, were tracked under diverse initial cell densities including OD_{700nm} 0.067, OD_{700nm} 0.078, OD_{700nm} 0.102, OD_{700nm} 0.148. Both the initial cell densities and co-culture growths were in Tecan Spark plate reader scale. The initial ratio of each member was 1:1:1 for these four co-cultures. N = three biologically independent samples, and data are presented as mean values +/- SD.

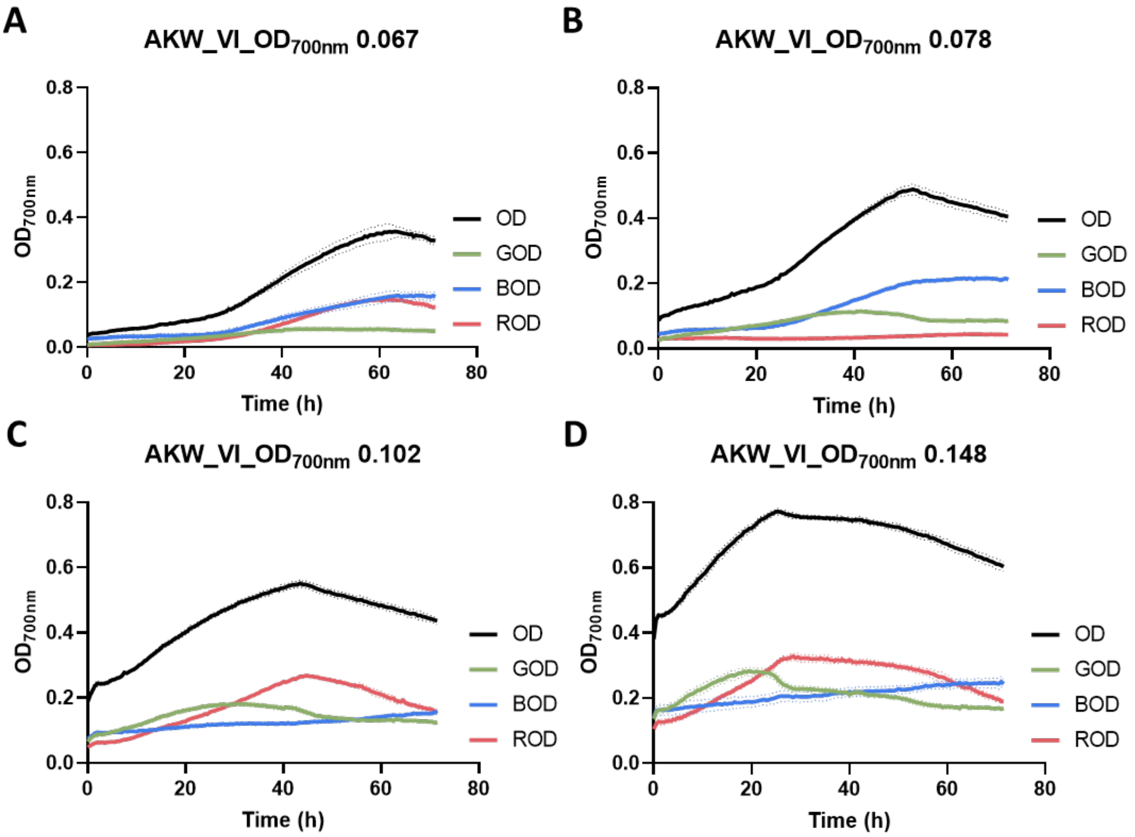

Fig. S28 Time courses of cell growth of co-culture AKW_VI and its individual member under different initial cell densities.

A-D. Time courses of cell growth of co-culture AKW_VI and its individual member under different initial cell densities, including OD_{700nm} 0.067, OD_{700nm} 0.078, OD_{700nm} 0.102, OD_{700nm} 0.148. Both the initial cell densities and co-culture growths were in Tecan Spark plate reader scale. The initial ratio of each member was 1:1:1 for these four co-cultures. GOD, BOD and ROD are estimated cell density tagged with GFP, BFP and RFP respectively, which were calculated using the standard curves between OD values and fluorescence intensities (GFP, BFP and RFP). N = three biologically independent samples, and data are presented as mean values +/- SD.

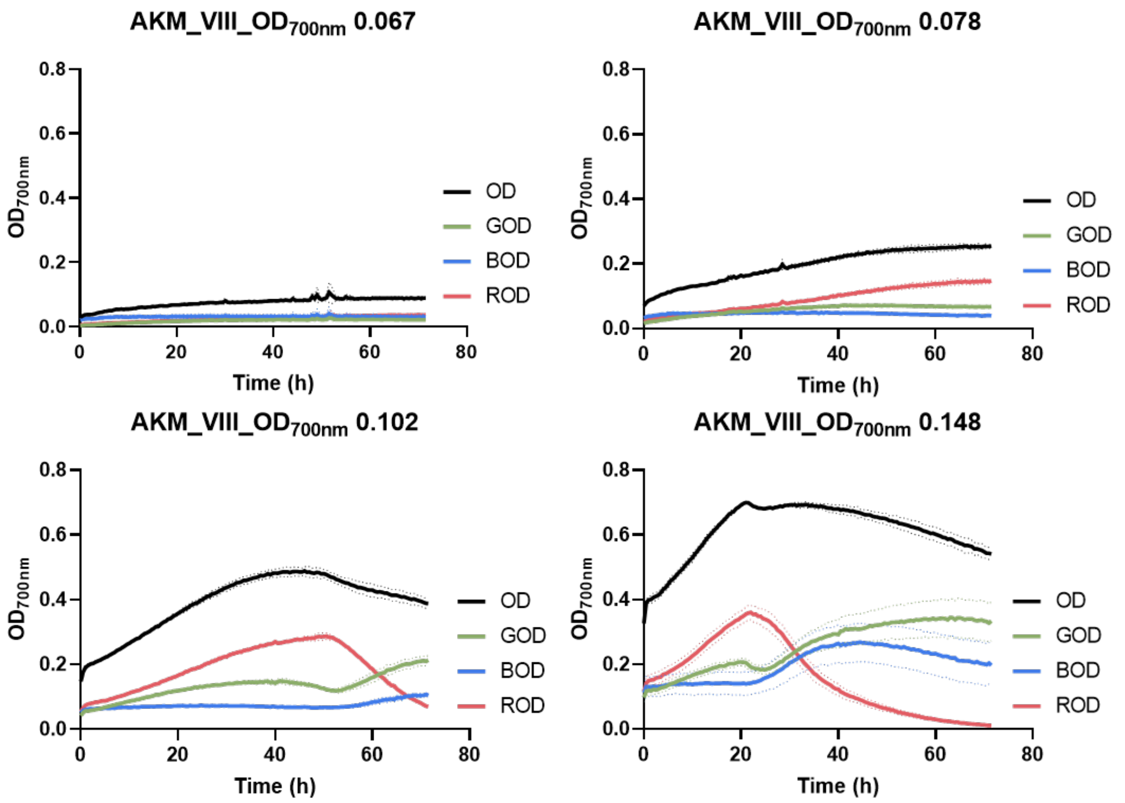

Fig. S29 Time courses of cell growth of co-culture AKH_VIII and its individual member under different initial cell densities.

A-D. Time courses of cell growth of co-culture AKH_VIII and its individual member under different initial cell densities, including OD$_{700nm}$ 0.067, OD$_{700nm}$ 0.078, OD$_{700nm}$ 0.102, OD$_{700nm}$ 0.148. Both the initial cell densities and co-culture growths were in Tecan Spark plate reader scale. The initial ratio of each member was 1:1:1 for these four co-cultures. GOD, BOD and ROD are estimated cell density tagged with GFP, BFP and RFP respectively, which were calculated using the standard curves between OD values and fluorescence intensities (GFP, BFP and RFP). N = three biologically independent samples, and data are presented as mean values +/- SD.

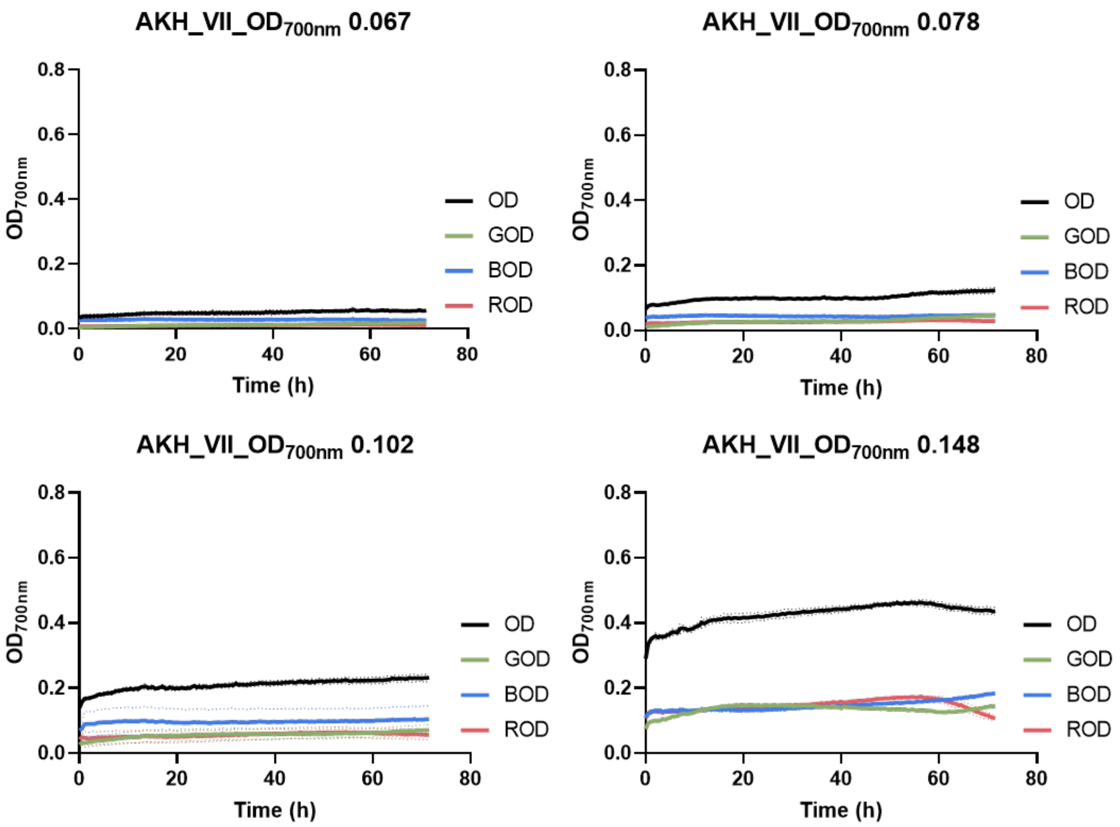

Fig. S30 Time courses of cell growth of co-culture AKH_VII and its individual member under different initial cell densities.

A-D. Time courses of cell growth of co-culture AKH_VII and its individual member under different initial cell densities, including OD_{700nm} 0.067, OD_{700nm} 0.078, OD_{700nm} 0.102, OD_{700nm} 0.148. Both the initial cell densities and co-culture growths were in Tecan Spark plate reader scale. The initial ratio of each member was 1:1:1 for these four co-cultures. GOD, BOD and ROD are estimated cell density tagged with GFP, BFP and RFP respectively, which were calculated using the standard curves between OD values and fluorescence intensities (GFP, BFP and RFP). N = three biologically independent samples, and data are presented as mean values +/- SD.

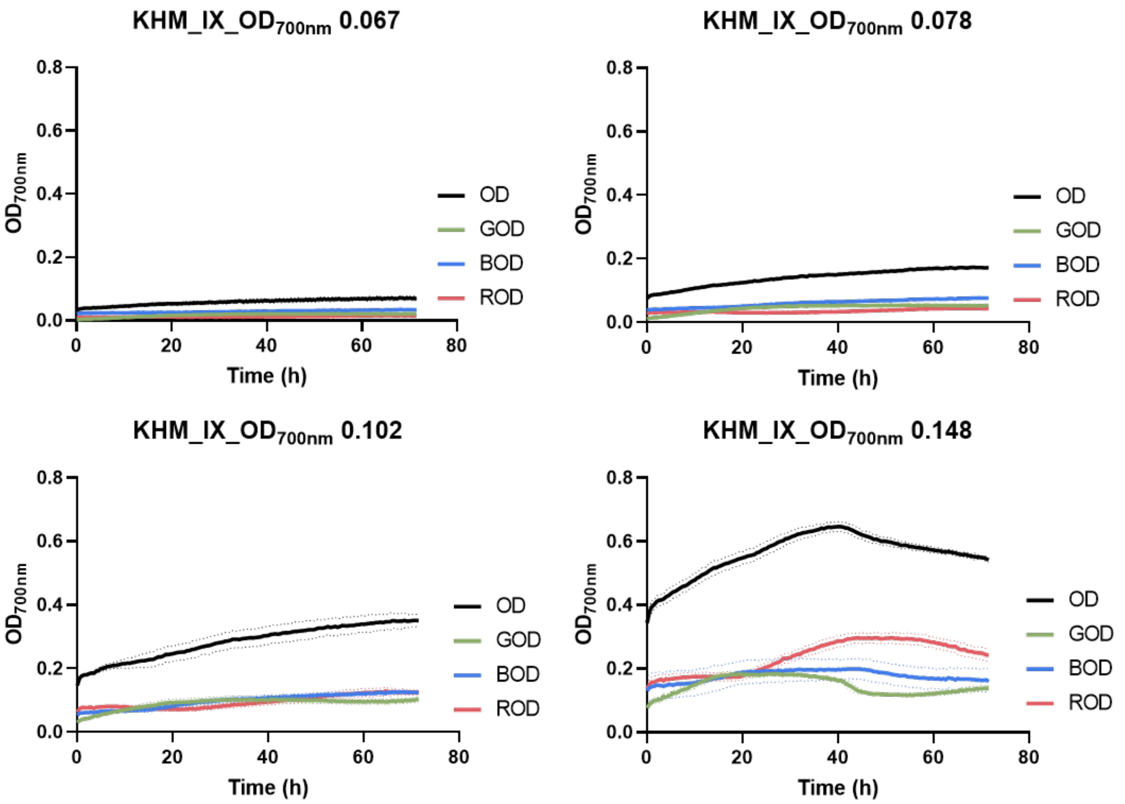

Fig. S31 Time courses of cell growth of co-culture AHM_IX and its individual member under different initial cell densities.

A-D. Time courses of cell growth of co-culture AHM_IX and its individual member under different initial cell densities, including OD_{700nm} 0.067, OD_{700nm} 0.078, OD_{700nm} 0.102, OD_{700nm} 0.148. Both the initial cell densities and co-culture growths were in Tecan Spark plate reader scale. The initial ratio of each member was 1:1:1 for these four co-cultures. GOD, BOD and ROD are estimated cell density tagged with GFP, BFP and RFP respectively, which were calculated using the standard curves between OD values and fluorescence intensities (GFP, BFP and RFP). N = three biologically independent samples, and data are presented as mean values +/- SD.

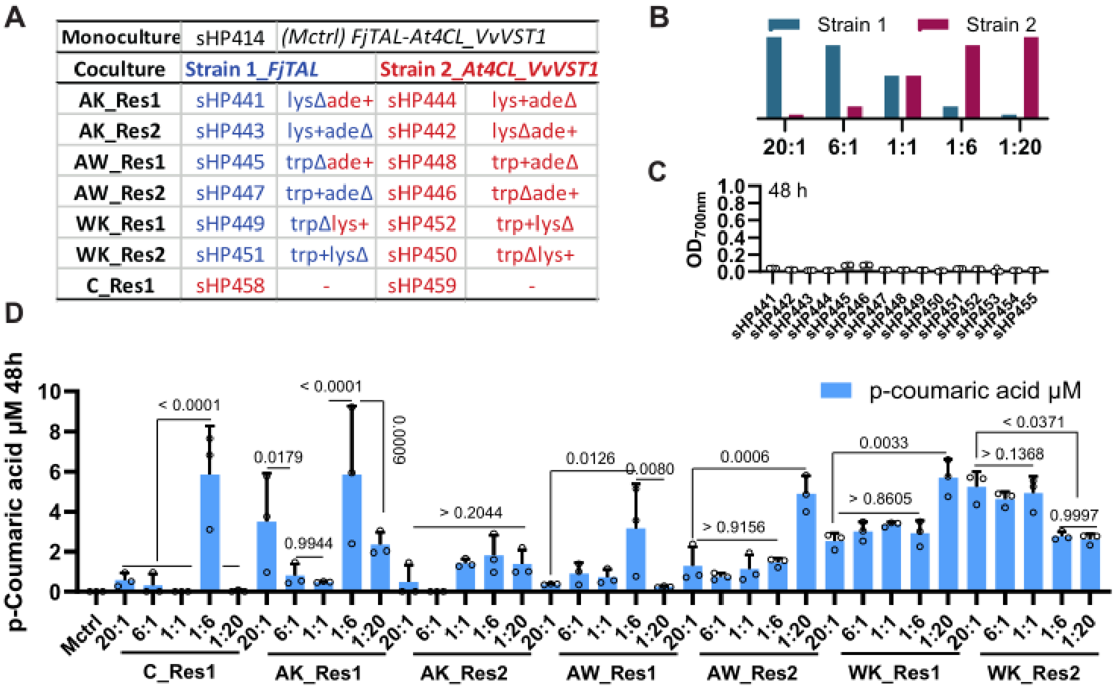

Monoculture	sHP414	(Mctrl) FjTAL-At4CL_VvVST1		
Coculture	Strain 1_FjTAL		Strain 2_At4CL_VvVST1	
AK_Res1	sHP441	lysΔade+	sHP444	lys+adeΔ
AK_Res2	sHP443	lys+adeΔ	sHP442	lysΔade+
AW_Res1	sHP445	trpΔade+	sHP448	trp+adeΔ
AW_Res2	sHP447	trp+adeΔ	sHP446	trpΔade+
WK_Res1	sHP449	trpΔlys+	sHP452	trp+lysΔ
WK_Res2	sHP451	trp+lysΔ	sHP450	trpΔlys+
C_Res1	sHP458	-	sHP459	-

Fig. S32 Co-culture strain setup and remaining p-coumaric acid concentrations in synthetic co-cultures for resveratrol production.

A. Strain table of monoculture control and 7 pairs of co-cultures for resveratrol production, including C_Res1 (WT), AK_Res1, 2 (2x ade-lys), AW_Res1, 2 (2x trp-ade) and WK_Res1, 2 (2x trp-lys). **B.** Different initial ratios were used for these co-culture setups, including 20:1, 6:1, 1:1, 1:6 and 1:20. **C.** OD_{700nm} at 48 h of negative controls of 12 auxotrophic monocultures used for 6 pairs of cross-feeding co-cultures in synthetic minimal medium. **D**. p-coumaric acid concentrations of 7 pairs of co-cultures and control of monoculture (Mctrl, sHP414) for resveratrol production at 48 h in synthetic minimal medium. N = three biologically independent samples, and data are presented as mean values +/- SD. Two-way ANOVA, followed by Bonferroni's multiple comparisons test with 95% confidence intervals were performed using Prism 9.5.0 (GraphPad) software, and p values were noted

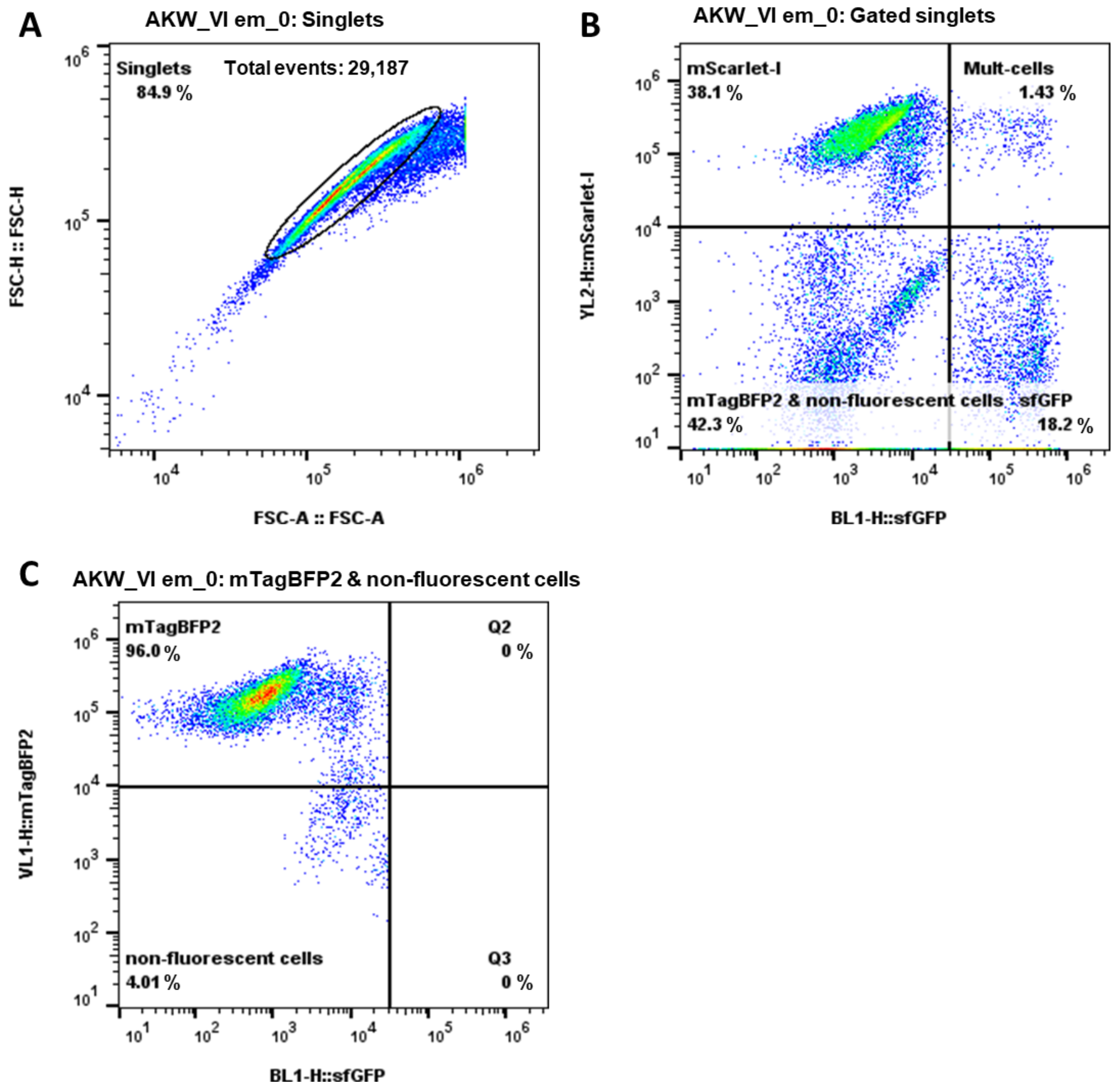

Fig. S33 Gating strategy for flow cytometry data

Sample co-culture AKW_VI without exchanged metabolite supplementation (AKW_VI em_0, Figure 5 F) was selected as an example to demonstrate the data gating strategy. **A.** Yeast cells were gated for singlets using FSC-H vs FSC-A to remove background noise. 29,187 (> 10,000) events were collected and analysed within the singlets gate for each measurement. **B.** Quadrant gating was applied in the double-fluorescence dimension (BL1-H::sfGFP vs YL2-H::mScarlet-I) to separate the population tagged with different fluorescent proteins including mscarlet-I (38.1%), sfGFP (18.2%), mTagBFP2 and non-fluorescent cells (42.3%), and multiple cells (1.43%) such as doublets and triplets. **C.** Quadrant gating was further applied in double-fluorescence dimension (BL1-H::sfGFP vs VL 1-H::mTagBFP2) to separate the population tagged with mTagBFP2 (96% out of 42.3%) and non-fluorescent cells (4% out of 42.3%). The percentage of mTagBFP2 tagged population was 96.0% x 42.3% = 38.98% and the percentage of non-fluorescent cells was 4.01% x 42.3% = 1.69%.
