# Supplementary material for: A molecular toolkit of cross-feeding strains for engineering synthetic yeast communities
Source: Nat Microbiol. 2024 Feb 7;9(3):848–63. doi: 10.1038/s41564-023-01596-4 (PMC10914607; doi:10.1038/s41564-023-01596-4)
Supplement: Supplementary file 2 — Reporting Summary [file 41564_2023_1596_MOESM2_ESM.pdf]

nature portfolio

Corresponding author(s): Rodrigo Ledesma-Amaro
Last updated by author(s): Dec 6, 2023

Reporting Summary

Nature Portfolio wishes to improve the reproducibility of the work that we publish. This form provides structure for consistency and transparency in reporting. For further information on Nature Portfolio policies, see our Editorial Policies and the Editorial Policy Checklist.

Statistics

For all statistical analyses, confirm that the following items are present in the figure legend, table legend, main text, or Methods section.

n/a	Confirmed	
☐	☒	The exact sample size (*n*) for each experimental group/condition, given as a discrete number and unit of measurement
☐	☒	A statement on whether measurements were taken from distinct samples or whether the same sample was measured repeatedly
☐	☒	The statistical test(s) used AND whether they are one- or two-sided<br>*Only common tests should be described solely by name; describe more complex techniques in the Methods section.*
☒	☐	A description of all covariates tested
☐	☒	A description of any assumptions or corrections, such as tests of normality and adjustment for multiple comparisons
☐	☒	A full description of the statistical parameters including central tendency (e.g. means) or other basic estimates (e.g. regression coefficient) AND variation (e.g. standard deviation) or associated estimates of uncertainty (e.g. confidence intervals)
☐	☒	For null hypothesis testing, the test statistic (e.g. *F*, *t*, *r*) with confidence intervals, effect sizes, degrees of freedom and *P* value noted<br>*Give P values as exact values whenever suitable.*
☒	☐	For Bayesian analysis, information on the choice of priors and Markov chain Monte Carlo settings
☒	☐	For hierarchical and complex designs, identification of the appropriate level for tests and full reporting of outcomes
☒	☐	Estimates of effect sizes (e.g. Cohen's *d*, Pearson's *r*), indicating how they were calculated

Our web collection on statistics for biologists contains articles on many of the points above.

Software and code

Policy information about availability of computer code

Data collection

1. Magellan Standard was used to collect data from the Tecan Spark plate reader.
2. Attune NxT software v3.1 was used to collect flow cytometry data.
3. Agilent MassHunt version 10 was used to collect LC-MS data.

Data analysis

1. GraphPad Prism 9 was used for generating most graphs and statistical analysis for wet lab experiments.
2. FlowJo v10.6.2 was used to analyse flow cytometry data.
3. Microsoft Excel was used to calculate the changes of cell growth OD values, fluorescent intensities, metabolite concentrations.
4. Benchling was used for designing all nucleotide sequences and CRISPR experiments.
5. MassHunter Quantitative software version 10 was used to analyse LC-MS data.
6. All simulations and related statistical analysis were carried out in MATLAB 2019a or MATLAB 2021a (both Mathworks Inc, MA, USA) using the in-built stiff solver ode15s. The global sensitivity toolbox, implemented in MATLAB, developed by Marino et al. was retrieved from http://malthus.micro.med.umich.edu/lab/usadata/ and used as detailed in their original publication.
7. Inkscape v1.2 was used to draw diagrams and assemble figures.

For manuscripts utilizing custom algorithms or software that are central to the research but not yet described in published literature, software must be made available to editors and reviewers. We strongly encourage code deposition in a community repository (e.g. GitHub). See the Nature Portfolio guidelines for submitting code & software for further information.

Data

Policy information about availability of data

All manuscripts must include a data availability statement. This statement should provide the following information, where applicable:

- Accession codes, unique identifiers, or web links for publicly available datasets
- A description of any restrictions on data availability
- For clinical datasets or third party data, please ensure that the statement adheres to our policy

-All source data are publicly available, either provided as (supplementary) source data with this paper, or published in public repository. Raw flow cytometry data for Fig. 4 & 5 are available on https://github.com/hdpeng89/Raw-flow-cytometry-data-yeast-co-culture.
-The MATLAB source code (with exemplar analysis runs as .mat files) was provided (with exemplar analysis runs as .mat files) on the Zendoo repository (DOI: 10.5281/zenodo.10257825).

Research involving human participants, their data, or biological material

Policy information about studies with human participants or human data. See also policy information about sex, gender (identity/presentation), and sexual orientation and race, ethnicity and racism.

Reporting on sex and gender	N/A
Reporting on race, ethnicity, or other socially relevant groupings	N/A
Population characteristics	N/A
Recruitment	N/A
Ethics oversight	N/A

Note that full information on the approval of the study protocol must also be provided in the manuscript.

Field-specific reporting

Please select the one below that is the best fit for your research. If you are not sure, read the appropriate sections before making your selection.

☒ Life sciences ☐ Behavioural & social sciences ☐ Ecological, evolutionary & environmental sciences

For a reference copy of the document with all sections, see nature.com/documents/nr-reporting-summary-flat.pdf

Life sciences study design

All studies must disclose on these points even when the disclosure is negative.

Sample size	No sample size calculation was performed, experiments were performed in triplicates (n = 3) or greater, which is the generally accepted by the scientific community.
Data exclusions	No data were excluded from the manuscript.
Replication	All experiments were performed in triplicates or greater and all attempts at replication were successful (sample size indicated in figure legend).
Randomization	Transformed yeast colonies were chosen at random from plates and no data was excluded.
Blinding	The study does not contain experiments where blinding would be applicable.

Reporting for specific materials, systems and methods

We require information from authors about some types of materials, experimental systems and methods used in many studies. Here, indicate whether each material, system or method listed is relevant to your study. If you are not sure if a list item applies to your research, read the appropriate section before selecting a response.

Materials & experimental systems

n/a	Involved in the study
☒	☐ Antibodies
☐	☒ Eukaryotic cell lines
☒	☐ Palaeontology and archaeology
☒	☐ Animals and other organisms
☒	☐ Clinical data
☒	☐ Dual use research of concern
☒	☐ Plants

Methods

n/a	Involved in the study
☒	☐ ChIP-seq
☐	☒ Flow cytometry
☒	☐ MRI-based neuroimaging

Eukaryotic cell lines

Policy information about cell lines and Sex and Gender in Research

Cell line source(s)	Saccharomyces cerevisiae Strain BY4741 from ATCC.
Authentication	We confirmed all derivative strains by colony PCR and sequencing.
Mycoplasma contamination	Yeast does not have this contamination.
Commonly misidentified lines (See ICLAC register)	No common misidentified lines were used.

Flow Cytometry

Plots

Confirm that:

☒ The axis labels state the marker and fluorochrome used (e.g. CD4-FITC).

☒ The axis scales are clearly visible. Include numbers along axes only for bottom left plot of group (a 'group' is an analysis of identical markers).

☒ All plots are contour plots with outliers or pseudocolor plots.

☒ A numerical value for number of cells or percentage (with statistics) is provided.

Methodology

Sample preparation	Cell cultures were diluted 4-5 dilutions using the culture synthetic minimal medium before measurement.
Instrument	Attune NxT3 Colour with Autosampler
Software	Attune NxT software for collection. FlowJo version 10.6 for data analysis.
Cell population abundance	Typical samples included at least 10,000 cells.
Gating strategy	Yeast cells were gated fro singlets using FSC-H vs FSC-A and to remove background noicse. No other Gatings was performed on global yeast population. > 10, 000 events were collected and analysed within the singlets gate fro each measurement.

☒ Tick this box to confirm that a figure exemplifying the gating strategy is provided in the Supplementary Information.
